# A homologous or variant booster vaccine after Ad26.COV2.S immunization enhances SARS-CoV-2–specific immune responses in rhesus macaques

**DOI:** 10.1126/scitranslmed.abm4996

**Published:** 2022-03-30

**Authors:** Xuan He, Malika Aid, Abishek Chandrashekar, Jingyou Yu, Katherine McMahan, Frank Wegmann, Catherine Jacob-Dolan, Jenny S. Maron, Caroline Atyeo, Huahua Wan, Daniel Sellers, Jinyan Liu, Michelle Lifton, Sarah Gardner, Esther A. Bondzie, Julia Barrett, Kunza Ahmad, Tochi Anioke, Jake Yalley-Ogunro, Jeanne Muench, Adrienne Goode, Hanne Andersen, Mark G. Lewis, Galit Alter, Hanneke Schuitemaker, Roland Zahn, Dan H. Barouch

**Affiliations:** 1 Center for Virology and Vaccine Research, Beth Israel Deaconess Medical Center, Harvard Medical School, Boston, MA 02215, USA.; 2 Janssen Vaccines and Prevention, Leiden, Netherlands.; 3 Harvard Medical School, Boston, MA 02115, USA.; 4 Ragon Institute of MGH, MIT and Harvard, Cambridge, MA 02139, USA.; 5 BIOQUAL, Rockville, MD 20852, USA.; 6 Amsterdam University Medical Center, Amsterdam, Netherlands.

## Abstract

Ad26.COV2.S has demonstrated durability and clinical efficacy against symptomatic COVID-19 in humans. In this study, we report the correlates of durability of humoral and cellular immune responses in 20 rhesus macaques immunized with single-shot Ad26.COV2.S and the immunogenicity of a booster shot at 8 to 10 months after the initial immunization. Ad26.COV2.S elicited durable binding and neutralizing antibodies as well as memory B cells and long-lived bone marrow plasma cells. Innate immune responses and bone marrow plasma cell responses correlated with durable antibody responses. After Ad26.COV2.S boost immunization, binding and neutralizing antibody responses against multiple SARS-CoV-2 variants increased 31- to 69-fold and 23- to 43-fold, respectively, compared with preboost concentrations. Antigen-specific B cell and T cell responses also increased substantially after the boost immunization. Boosting with a modified Ad26.COV2.S.351 vaccine expressing the SARS-CoV-2 spike protein from the beta variant led to largely comparable responses with slightly higher beta- and omicron-specific humoral immune responses. These data demonstrate that a late boost with Ad26.COV2.S or Ad26.COV2.S.351 resulted in a marked increase in humoral and cellular immune responses that were highly cross-reactive across multiple SARS-CoV-2 variants in rhesus macaques.

## The value of variant boosters

Ad26.COV2.S is an approved vaccine for SARS-CoV-2 and has been shown to elicit protective and durable immune responses. However, as boosters are now considered part of a full immunization regimen, the response to homologous boosting with Ad26.COV2.S merits study. Here, He *et al.* evaluated humoral and cellular immune responses in nonhuman primates vaccinated and boosted with Ad26.COV2.S, showing that boosting increased both responses. Further, boosting with a modified Ad26.COV2.S.351, which uses the spike protein from the SARS-CoV-2 beta variant, elicited increased immune responses that were cross-reactive, including against the omicron variant. Together, these data provide evidence that homologous or variant-specific booster immunizations may further potentiate the effectiveness of Ad26.COV2.S.

## Teaser

Ad26.COV2.S vaccination generated durable immune responses that were enhanced by homologous or beta variant boosters in rhesus macaques.

## INTRODUCTION

The Ad26.COV2.S vaccine is a replication-incompetent adenovirus (Ad) 26 vector (*1*) expressing the stabilized severe acute respiratory syndrome coronavirus 2 (SARS-CoV-2) spike protein (*2*) from the Wuhan 2019 strain, which is identical to the spike protein from the WA1/2020 strain. Immunogenicity and protective efficacy of the single-shot Ad26.COV2.S vaccine have been demonstrated in hamsters and rhesus macaques (*3*–*6*) as well as in humans (*4*, *7*–*10*). Recent data have also shown durability of immune responses induced by single-shot Ad26.COV2.S in humans for at least 8 months (*11*). However, waning immunity has also been reported for coronavirus disease 2019 (COVID-19) vaccines (*12*), and recent reports of breakthrough infections with the SARS-CoV-2 variants, including delta (B.1.617.2) and omicron (B.1.1.529), in fully vaccinated individuals (*13*–*16*) have subsequently highlighted the need for boost immunizations for all the currently approved vaccines, including Ad26.COV2.S.

Moreover, immune imprinting has been observed after both COVID-19 infection (*17*) and immunizations (*18*). By immune imprinting, the initial COVID-19 vaccination might shape the subsequent immune responses against variants of concern after homologous or heterologous boosts. In this study, we evaluated the correlates of durability of single-shot Ad26.COV2.S vaccination in rhesus macaques and the immunogenicity of a late boost at 8 to 10 months with Ad26.COV2.S or Ad26.COV2.S.351, which expresses the stabilized SARS-CoV-2 spike protein from the B.1.351 (beta) variant.

## RESULTS

### Durability of immune responses after single-shot Ad26.COV2.S vaccination

**Fig. 1.**
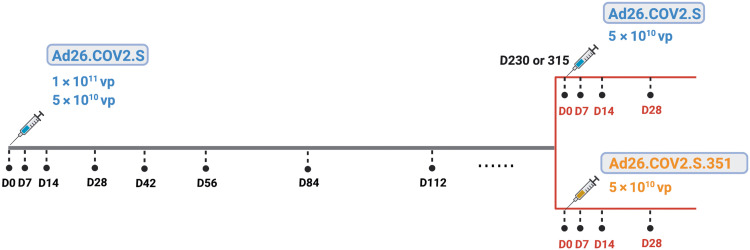
Study schema. Twenty rhesus macaques were immunized with 1 × 10^11^ vp (*n* = 10) or 5 × 10^10^ vp (*n* = 10) Ad26.COV2.S. The study start was staggered, and half of the animals in each dose group were followed for 230 or 315 days. Blood was collected longitudinally from days 0 to day 230 or 315 after immunization to evaluate the durability of single-dose Ad26.COV2.S. On day 230 or 315, all the macaques received a boost immunization with 5 × 10^10^ vp Ad26.COV2.S (*n* = 10) or Ad26.COV2.S.351 (*n* = 10). vp, viral particles; D, day.

**Fig. 2.**
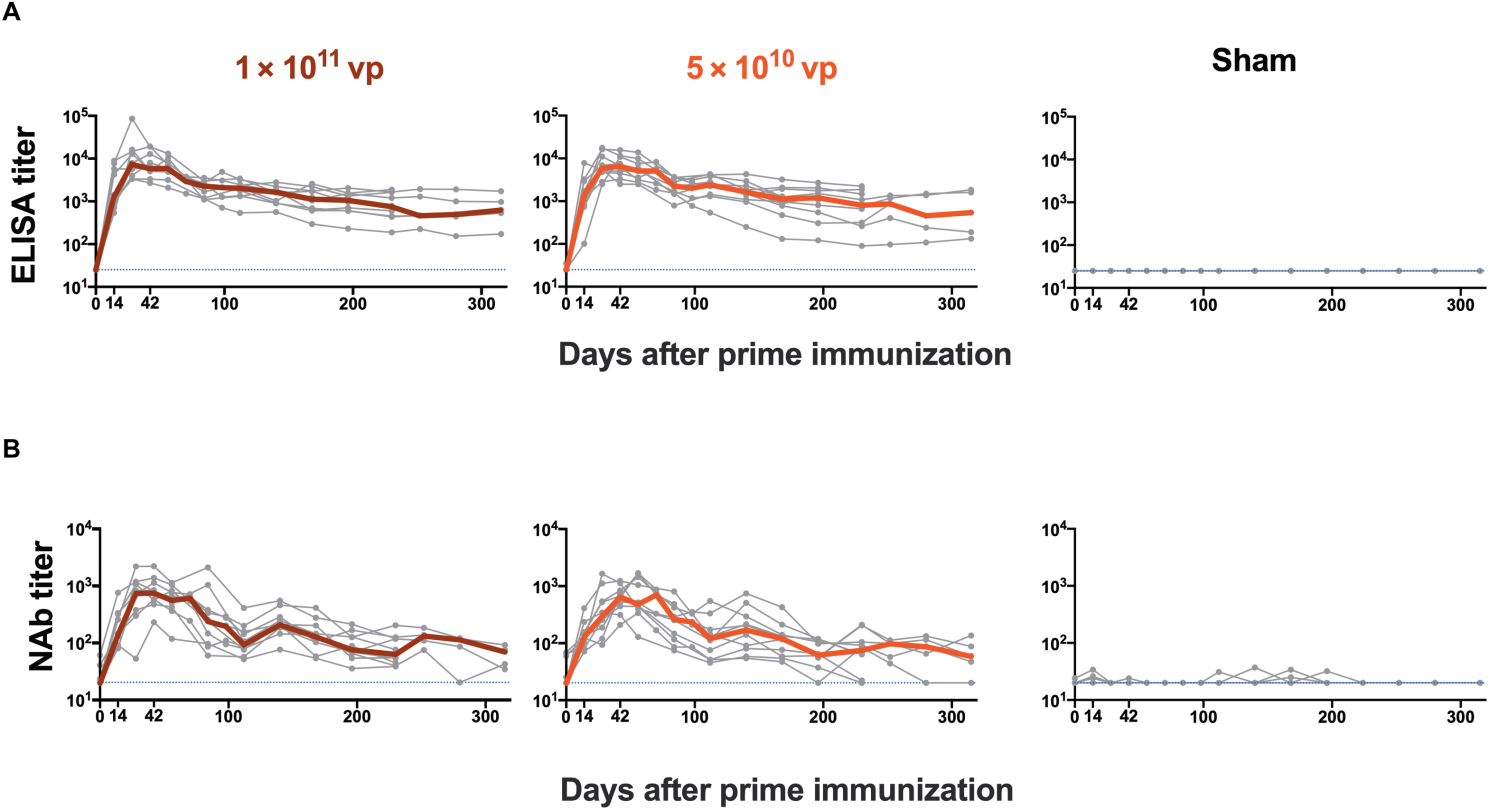
SARS-CoV-2–specific antibody responses are durable after single-shot Ad26.COV2.S vaccination. (**A**) SARS-CoV-2 WA1/2020 RBD-specific binding antibody responses were measured by ELISA against SARS-CoV-2 in rhesus macaques after single-shot immunization with 1 × 10^11^ vp (*n* = 10) or 5 × 10^10^ vp (*n* = 10) Ad26.COV2.S or sham (*n* = 4). (**B**) Pseudovirus neutralizing antibody (NAb) responses against SARS-CoV-2 WA1/2020 were measured. Bold lines reflect median values. Dotted lines reflect lower limits of quantitation.

We first assessed the durability of humoral and cellular immune responses after single-shot Ad26.COV2.S vaccination. Twenty rhesus macaques were immunized by the intramuscular route with 10^11^ viral particles (vp) (*n* = 10) or 5 × 10^10^ vp (*n* = 10) of Ad26.COV2.S and were followed for either 230 or 315 days, which reflected a staggered start for half of the animals in each dose group (Fig. 1). An additional four animals were included as sham controls. Receptor binding domain (RBD)–specific binding antibody responses were assessed by enzyme-linked immunosorbent assay (ELISA; Fig. 2A) (*4*, *19*, *20*). WA1/2020 RBD-specific ELISA responses peaked on days 28 to 56 at median titers of 7413 [interquartile range (IQR), 3802 to 14,835) and 6478 (IQR, 4173 to 10,696) with the 10^11^ vp and 5 × 10^10^ vp doses, respectively, and then showed a biphasic decay with half-lives of 38 days and 139 to 254 days, respectively (fig. S1). All animals showed binding antibody responses for the duration of follow-up with no difference between the two doses tested. Neutralizing antibody (NAb) responses against SARS-CoV-2 WA1/2020 were assessed by a luciferase-based pseudovirus NAb assay (*4*, *19*–*21*) and showed similar kinetics to the binding antibody responses (Fig. 2B). NAb responses peaked on days 28 to 56 at median titers of 763 (IQR, 534 to 1179) and 649 (IQR, 408 to 904) with the 10^11^ vp and 5 × 10^10^ vp doses, respectively, and then showed a biphasic decay with half-lives of 30 to 44 days and 184 to 247 days (fig. S1). Seventeen of 20 animals showed NAb responses by days 230 to 315. Similar magnitudes and kinetics of antibody responses were observed for the two vaccine doses tested.

Spike (S) protein–specific cellular immune responses were assessed by pooled peptide enzyme-linked immune absorbent spot (ELISPOT) assays using overlapping 15–amino acid peptides in peripheral blood mononuclear cells (PBMCs). Interferon-γ (IFN-γ) ELISPOT responses to SARS-CoV-2 WA1/2020 spike protein peaked on day 14 and then declined gradually over 230 days (fig. S2). ELISPOT responses were similar for the two vaccine doses tested.

### Antigen-specific B cell and plasma cell responses

**Fig. 3.**
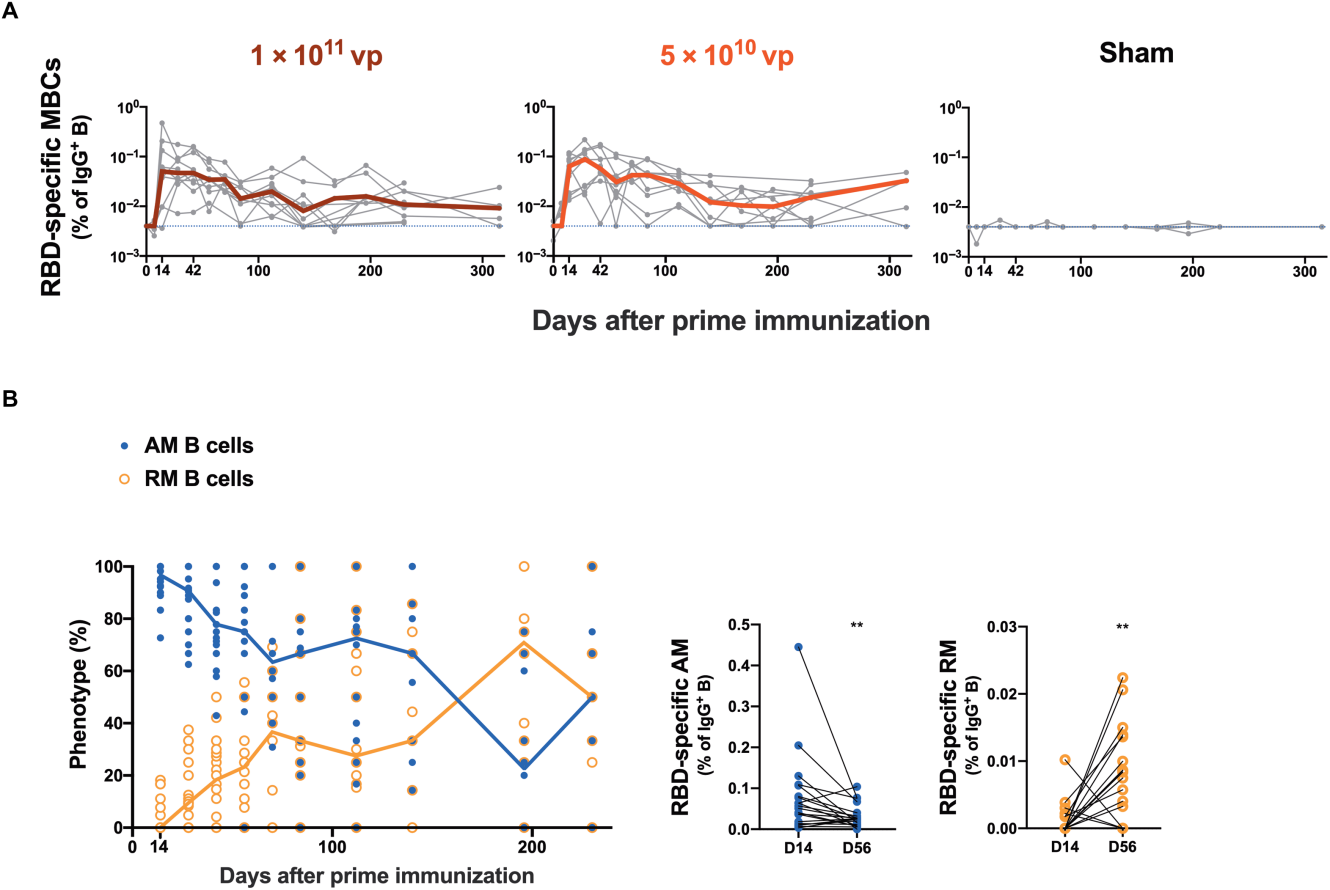
RBD-specific memory B cell responses are sustained after single-shot Ad26.COV2.S vaccination. (**A**) Longitudinal analysis of WA1/2020 RBD-specific IgG^+^ memory B cells (MBCs) in macaques is shown after single-shot immunization with 1 × 10^11^ vp (*n* = 10) or 5 × 10^10^ vp (*n* = 10) Ad26.COV2.S or sham (*n* = 4). Bold lines reflect median values. Dotted lines reflect lower limits of quantitation. (**B**) Longitudinal WA1/2020 RBD-specific AM (blue) and RM B cell (orange) proportions are shown after initial vaccination. Blue and orange lines reflect median values for AM and RM B cells, respectively. SARS-CoV-2 RBD-specific AM (blue) and RM (orange) B cells in IgG^+^ B cells are shown on days 14 and 56 after prime immunization. **P < 0.01, two-sided Wilcoxon signed-rank test.

Long-lived antigen-specific immunoglobulin G–positive (IgG^+^) memory B cells are important for generating anamnestic responses after antigen reexposure. WA1/2020 RBD-specific IgG^+^ memory B cells were assessed longitudinally in PBMCs by multiparameter flow cytometry (*6*). Ad26.COV2.S elicited RBD-specific memory B cells in all macaques by days 14 to 28 followed by a gradual decline, and specific B cell responses remained detectable in 17 of 20 animals by days 230 to 315 with no differences between the two doses tested (Fig. 3A). RBD-specific memory B cells initially exhibited an activated memory phenotype (AM; CD21^−^CD27^+^), which gradually transitioned into a resting memory phenotype (RM; CD21^+^CD27^+^) between days 14 and 230 after immunization (Fig. 3B and fig. S3). The frequencies of RBD-specific memory B cells on day 28 correlated with binding and NAb titers on day 28 (*P* < 0.0001, *R* = 0.7614 and *P* < 0.0001, *R* = 0.7306, respectively, two-sided Spearman rank correlation tests; fig. S4).

**Fig. 4.**
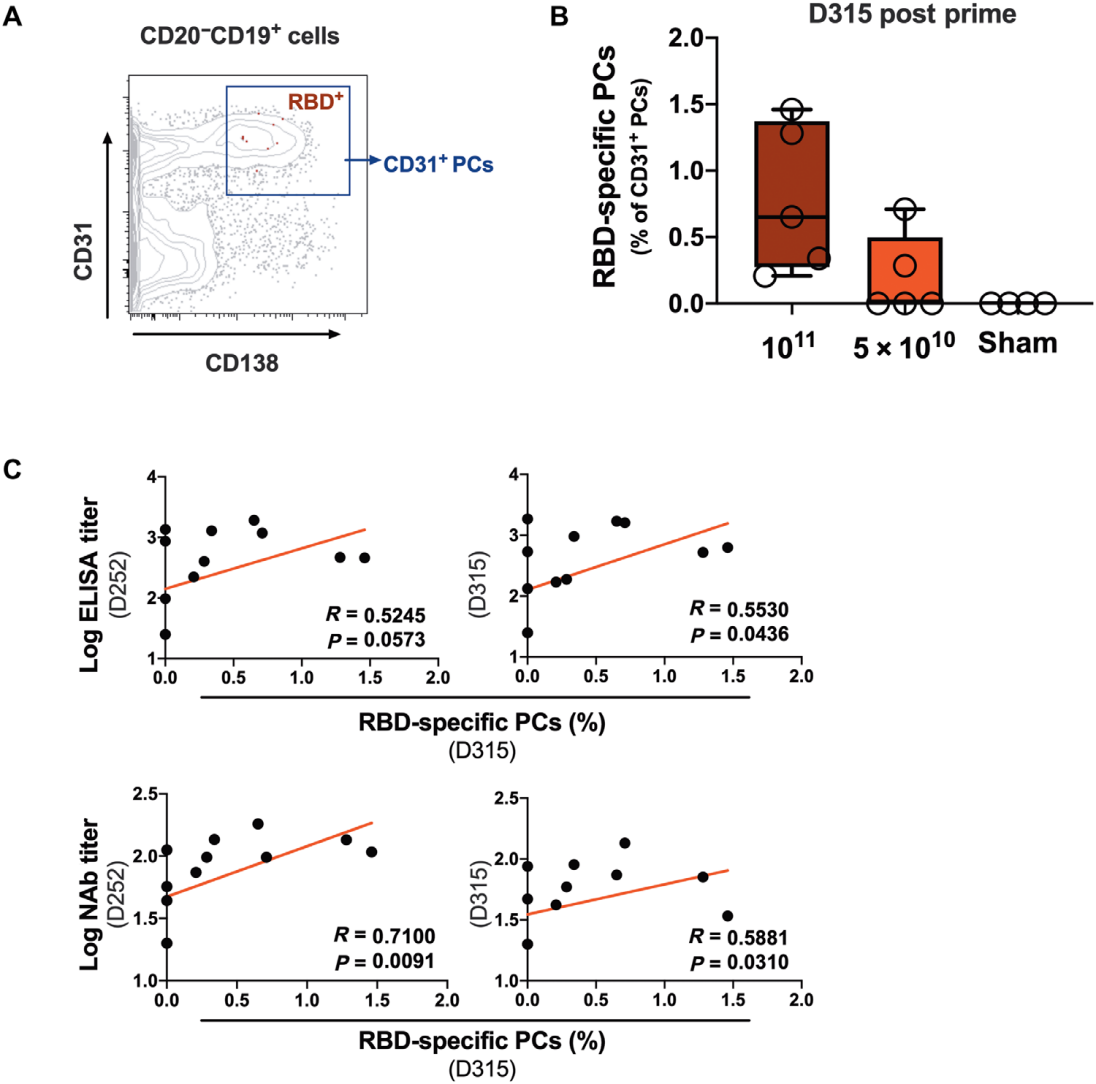
RBD-specific PCs in bone marrow persist after single-shot Ad26.COV2.S vaccination. (**A**) Representative flow cytometry plot show WA1/2020 RBD-specific plasma cells (PCs) in the CD138^+^CD31^+^ population gated from CD20^−^CD19^+^ cells in bone marrow isolated from vaccinated macaques. (**B**) Frequencies of RBD-specific CD31^+^ PCs in bone marrow in macaques on day 315 after single-shot immunization with 1 × 10^11^ vp (*n* = 5) or 5 × 10^10^ vp (*n* = 5) Ad26.COV2.S or sham (*n* = 4). Box and whisker plots indicate interquartile ranges (boxes), medians (horizontal lines), and range (whiskers). (**C**) Correlation of RBD-specific PCs on day 315 with binding (top panels) or neutralizing (bottom panels) antibody responses on day 252 (left panels) or day 315 (right panels) after vaccination. Red lines reflect best linear fits. *P* and *R* values reflect two-sided Spearman rank correlation tests.

Long-lived plasma cells (PCs) in the bone marrow are important for maintaining durable circulating antibodies (*22*–*24*). Bone marrow aspirates were collected from 10 vaccinated macaques on day 315 (half from each dose group) and from 4 unvaccinated sham control macaques. CD138^+^CD31^+^ PCs have been reported to be highly enriched for IgG-secreting B cells (*25*). We observed high expression of IgG and CD95 and low expression of Ki67 on CD138^+^CD31^+^ PCs, as expected (fig. S5). On day 315 after vaccination, bone marrow WA1/2020 RBD-specific PCs were detected in 7 of the 10 vaccinated macaques (Fig. 4, A and B). Bone marrow RBD-specific PCs correlated with NAb titers on day 252 (*P* = 0.0091, *R* = 0.7100, two-sided Spearman rank correlation tests) and with binding and NAb titers on day 315 (*P* = 0.0436, *R* = 0.5530 and *P* = 0.0310, *R* = 0.5881, respectively, two-sided Spearman rank correlation tests; Fig. 4C).

### Transcriptomic signatures of durability

**Fig. 5.**
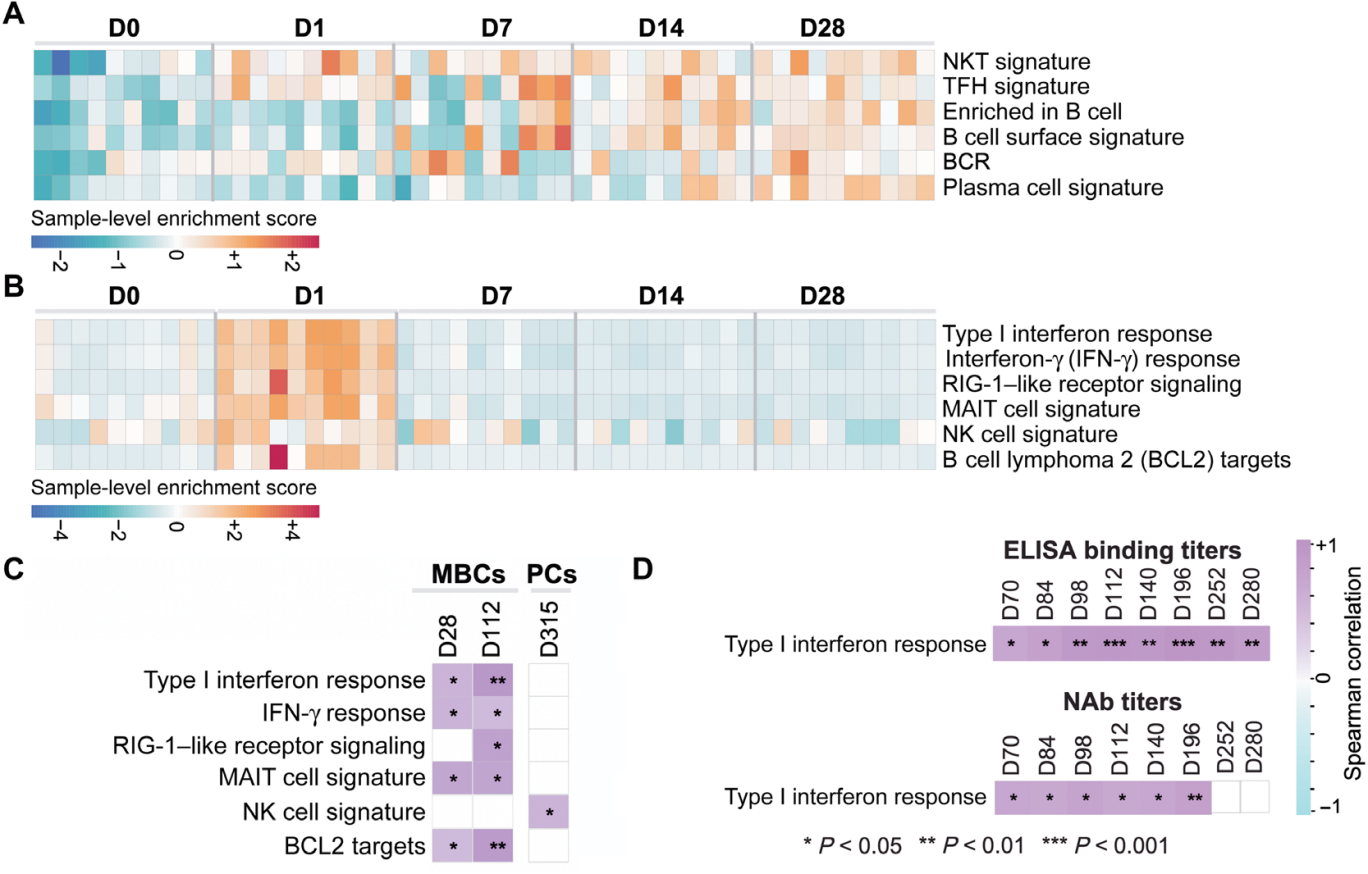
Innate immune signatures correlated with durable antibody responses. (**A**) Heatmaps show the row scaled sample-level enrichment (SLE) scores of natural killer T (NKT), T follicular helper (Tfh), B cell, BCR, and PC signatures on days 0, 1, 7, 14, and 28 after single-shot Ad26.COV2.S vaccination. (**B**) Heatmaps show the row scaled SLE scores of innate pathways up-regulated on day 1 after single-shot Ad26.COV2.S vaccination. (**C**) Correlation between SLE scores of pathways up-regulated on day 1 after single-shot Ad26.COV2.S vaccination and RBD-specific MBCs or PCs after vaccination. Columns correspond to individual animals, and rows correspond to individual pathways. Purple color gradient indicates the *Z* score–normalized SLE of each pathway across animals, measured as the mean expression of pathway leading genes (BH-adjusted *P* < 0.05) for each individual animal. (**D**) Spearman correlation of type I IFN signature SLE score and binding or neutralizing antibody titers is shown for multiple time points. Spearman correlation coefficients range from cyan (negative correlation) to purple (positive correlation). Blank squares indicate no significance. *P < 0.05, **P < 0.01, and ***P < 0.001.

To define transcriptomic signatures after Ad26.COV2.S vaccination and correlations with durable B cell and antibody responses, we performed bulk RNA sequencing of PBMCs on days 0, 1, 7, 14, and 28 in 10 animals that had sufficient cells after Ad26.COV2.S vaccination (half from each dose group). Gene set enrichment analyses (GSEA) revealed up-regulation of B cell pathways (Fig. 5A). Signatures of T follicular helper (Tfh) cells, B cells, B cell receptor (BCR) signaling, and PCs were up-regulated on days 7 to 28 [false discovery rate (FDR) *q* < 0.05], and signatures of natural killer T (NKT) cells were up-regulated on days 1 to 28 (FDR *q* < 0.05) after vaccination (Fig. 5A).

Innate pathways, including type I IFN responses, IFN-γ responses, retinoic acid-inducible gene I (RIG-I)-like receptor signaling, mucosal-associated invariant T (MAIT) cell signatures, natural killer (NK) cell signatures, and B cell lymphoma 2 (BCL2) survival signaling targets, were also up-regulated (FDR *q* < 0.05) on day 1 after vaccination and then rapidly resolved (Fig. 5B). These pathways correlated with RBD-specific memory B cell responses at both early (day 28) and late (day 112) time points (Fig. 5C). NK cell signatures on day 1 also correlated with long-term PC responses (day 315) (Fig. 5C). In addition, type I IFN signatures correlated with binding and NAb titers at multiple time points after immunization (Fig. 5D). These data show that early innate responses after vaccination correlated with durable B cell, PC, and antibody responses.

### Immunogenicity of Ad26.COV2.S or Ad26.COV2.S.351 boost immunization

**Fig. 6.**
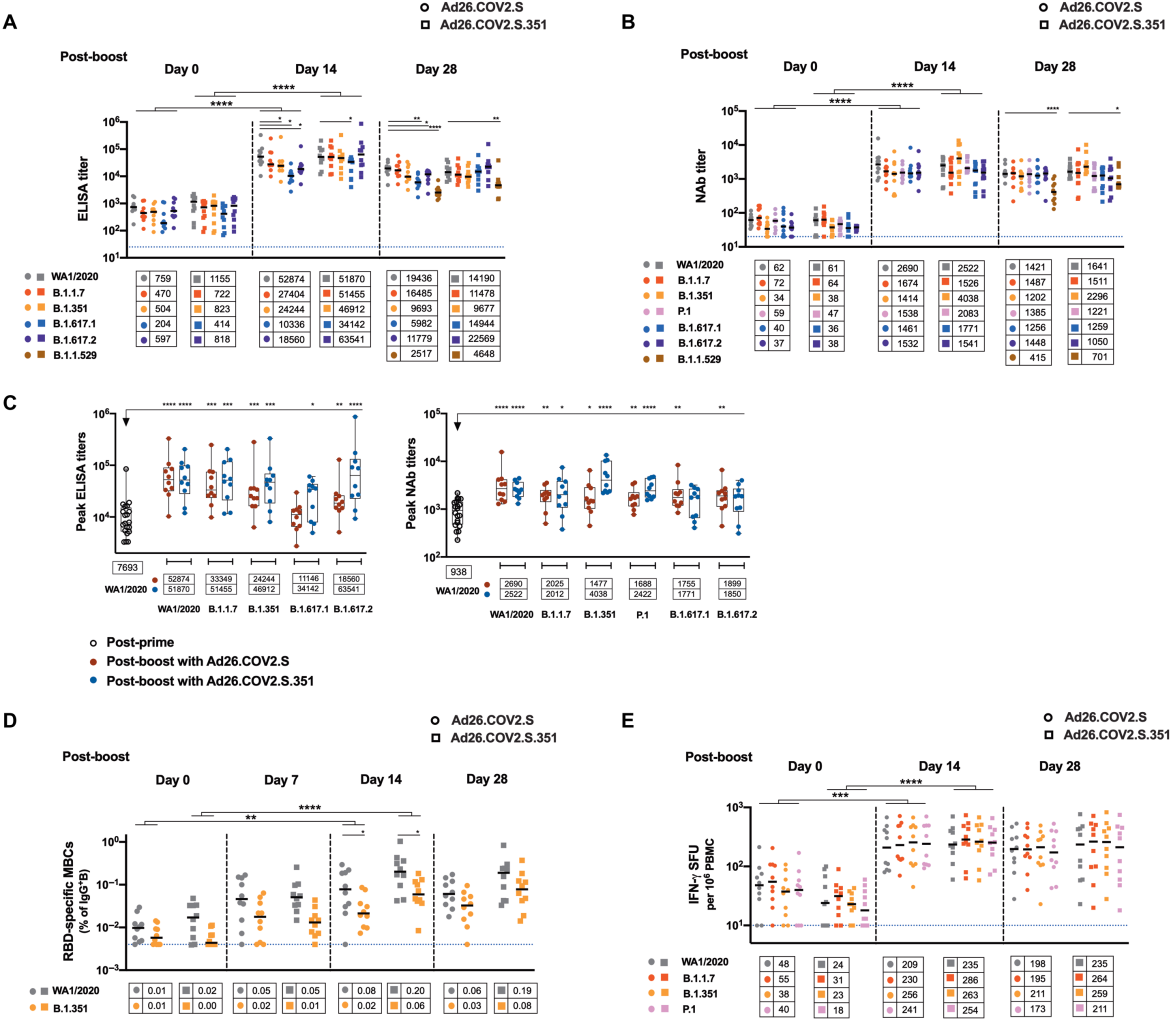
SARS-CoV-2–specific antibody and T cell responses are enhanced after boost immunization. (**A**) RBD-specific binding antibody responses and (**B**) NAb responses against SARS-CoV-2 variants WA1/2020 (ancestral), B.1.1.7 (alpha), B.1.351 (beta), P.1 (gamma), B.1.617.1 (kappa), and B.1.617.2 (delta) are shown for samples collected at days 0, 14, and 28 after the boost immunization with 5 × 10^10^ vp Ad26.COV2.S (*n* = 10) or Ad26.COV2.S.351 (*n* = 10). Responses to B.1.1.529 (omicron) are also shown for samples collected on day 28. Antibody responses to WA1/2020 were compared with responses to variants at each time point. Antibody responses against variants from day 0 were compared with responses from day 14 after the boost immunization. (**C**) Comparisons of peak responses against WA1/2020 after the initial single-shot Ad26.COV2.S immunization and peak responses against WA1/2020, B.1.1.7, B.1.351, P.1, B.1.617.1, and B.1.617.2 after the boost immunization with 5 × 10^10^ vp Ad26.COV2.S or Ad26.COV2.S.351 are shown. Box and whisker plots indicate interquartile ranges (boxes), medians (horizontal lines), and range (whiskers). (**D**) RBD-specific MBCs and (**E**) T cell responses by pooled peptide IFN-γ ELISPOT assays were measured on samples collected at days 0, 14, and 28 after the boost immunization. B cell responses to WA1/2020 were compared with responses to the beta variant at each time point. B cell and T cell responses against variants from day 0 were compared with responses from day 14 after the boost immunization. Bold horizontal lines and numbers in boxes reflect median values. Dotted lines reflect lower limits of quantitation. *P < 0.05, **P < 0.01, ***P < 0.001, and ****P < 0.0001, two-sided Mann-Whitney tests for comparisons between WA1/2020 group and individual variants at each time point (smaller asterisks), as well as for comparisons between individual variants on days 0 and 14 (larger asterisks reflecting *P* values for the individual variants compared between two groups). SFU, spot-forming unit.

All 20 vaccinated macaques received a boost immunization at 8 or 10 months after the initial immunization, which reflected the staggered start of the original study. Animals received a boost with 5 × 10^10^ vp Ad26.COV2.S (*n* = 10) or Ad26.COV2.S.351 (*n* = 10), which expresses the stabilized spike protein from the SARS-CoV-2 B.1.351 (beta) variant. The 5 × 10^10^ vp dose is the clinically approved dose for Ad26.COV2.S for both priming and boosting. Before the boost, binding and NAb responses against SARS-CoV-2 variants were observed with median ELISA titers of 278 (IQR, 135 to 776) to 812 (IQR, 553 to 1682) and median NAb titers of 37 (IQR, 31 to 41) to 67 (IQR, 48 to 119) (Fig. 6, A and B). By day 14 after the Ad26.COV2.S boost immunization, RBD-specific binding antibody responses increased 31- to 69-fold compared with preboost values against the ancestral (WA1/2020), alpha (B.1.1.7), beta (B.1.351), kappa (B.1.617.1), and delta (B.1.617.2) SARS-CoV-2 variants (*P* < 0.0001 for all, two-sided Mann-Whitney tests; Fig. 6A). By day 14, NAb responses increased 23- to 43-fold compared with preboost values against the ancestral, alpha, beta, gamma (P.1), kappa, and delta SARS-CoV-2 variants (*P* < 0.0001 for all, two-sided Mann-Whitney tests; Fig. 6B). Twenty-eight days after the boost immunization, NAb responses to the beta and delta variants were only 1.9- and 1.7-fold lower than the WA1/2020 response, respectively, after the boost immunization (*P* > 0.05, not significant for both, two-sided Mann-Whitney tests). At day 28 after boost immunization, binding and NAb responses against omicron were 7.7- and 3.4-fold lower than WA1/2020 responses, respectively, after boosting (*P* < 0.0001 for both, two-sided Mann-Whitney tests). WA1/2020-specific ELISA and NAb responses after the boost were 6.8- and 2.8-fold greater, respectively, than peak responses on days 28 to 56 after the initial single-shot Ad26.COV2.S immunization (*P* < 0.0001 and *P* < 0.0001, respectively, two-sided Mann-Whitney tests; Fig. 6C), suggesting that the boost immunization led to robust anamnestic antibody responses. Boosting with Ad26.COV2.S.351 resulted in largely comparable humoral immune responses, with 1.9- and 2.8-fold higher beta-specific NAb responses on days 14 and 28, respectively (*P* < 0.05 for both, two-sided Mann-Whitney tests), and with a 1.8-fold higher omicron-specific binding antibody response on day 28 (*P* < 0.05, two-sided Mann-Whitney tests) compared with Ad26.COV2.S boosting (Fig. 6, A and B).

An electrochemiluminescence assay (ECLA) (*26*) was also used to evaluate RBD- and S-specific binding antibody responses to multiple SARS-CoV-2 variants on day 28 and day 230/315 after initial immunization and on day 14 after the boost immunization and similarly demonstrated substantial boosting to all antigens tested (*P* < 0.0001 for all, two-sided Mann-Whitney tests; fig. S6). After the boost immunization, robust antibody-dependent cellular phagocytosis (ADCP) and antibody-dependent complement deposition (ADCD) (*10*, *27*) were also observed against multiple variants (fig. S7).

Boosting with Ad26.COV2.S or Ad26.COV2.S.351 led to rapid and robust B cell memory recall responses with a 4- to 10-fold increase in RBD-specific B cells at day 14 relative to day 0 (*P* < 0.01 for both, two-sided Mann-Whitney tests; Fig. 6D), which showed an activated memory B cell phenotype (fig. S8). Boost immunization also induced a 4- to 23-fold increase in T cell responses by IFN-γ ELISPOT assays at day 14 (*P* < 0.001 for both, two-sided Mann-Whitney tests; Fig. 6E). Humoral and cellular immune responses were largely comparable across all the variants studied after the boost immunization, suggesting substantial cross-reactivity against SARS-CoV-2 variants.

## DISCUSSION

The single-shot Ad26.COV2.S vaccine has demonstrated protective efficacy in humans in the United States, Latin America, and South Africa in the phase 3 ENSEMBLE study, including against the SARS-CoV-2 beta variant (*9*), as well as real-world effectiveness against severe disease from the SARS-CoV-2 delta and omicron variants. We recently reported durable humoral and cellular immune responses with minimal evidence of decline in humans over a period of 8 months after Ad26.COV2.S vaccination (*11*). However, increased breakthrough infections with the highly transmissible SARS-CoV-2 delta and omicron variants in fully vaccinated individuals have led to recommendations for booster shots for all the currently approved vaccines. A deeper understanding of the durability and benefit of boosting with Ad26.COV2.S is therefore needed.

In this study, we report the durability of single-shot Ad26.COV2.S vaccination in rhesus macaques and the immunogenicity of a boost immunization at 8 to 10 months. Durability of single-shot Ad26.COV2.S vaccination has also been reported in humans (*11*, *28*); after Ad26.COV2.S vaccination, neutralizing antibodies were threefold higher in humans than reported here in macaques (*28*). We also reported a NAb threshold for protection against SARS-CoV-2 challenge in a model of short-term natural immunity in macaques (*29*). In the present study, 14 of 20 macaques showed durable NAb titers above this threshold.

Here, we showed that single-shot Ad26.COV2.S vaccination induced durable antigen-specific memory B cells and bone marrow PCs. The frequencies of RBD-specific PCs residing in the bone marrow correlated with serum binding and neutralizing antibodies. In addition, we observed that early activation of IFN and inflammatory pathways correlated with durable B cell, PC, and antibody responses, reflecting the impact of innate immunity on humoral immune memory.

A boost immunization with Ad26.COV2.S or Ad26.COV2.S.351 in macaques led to a substantial 1 to 2 log increase in binding and NAb titers to values that were higher than peak responses after initial immunization, as well as markedly enhanced memory B cell and T cell responses. Moreover, humoral and cellular immune responses after the boost immunization were highly cross-reactive against multiple SARS-CoV-2 variants, including the alpha, beta, gamma, and delta variants, with only minimal evidence for immune imprinting. Humoral responses against the omicron variant were lower, consistent with published data (*13*–*15*). Boosting with Ad26.COV2.S.351 led to modestly higher beta-specific and omicron-specific humoral immune responses. Moreover, the efficiency of the observed boosting suggests that anti-vector immunity after initial immunization (*8*) does not preclude a second immunization with homologous Ad26.COV2.S or heterologous Ad26.COV2.S.351. Although the mechanisms of protection against SARS-CoV-2 in humans remain to be determined, we speculate that the substantially enhanced immune responses after boost immunization will lead to improved protective efficacy. We previously demonstrated that both antibodies and T cells in convalescent rhesus macaques contributed to protection against SARS-CoV-2 challenge (*29*).

Boosting humans at 6 months with Ad26.COV2.S has been shown to result in a ninefold increase in antibody titers (*30*), whereas boosting humans at 2 months led to a three- to fourfold increase in antibody titers (*7*). Similarly, the boost observed in macaques at 8 to 10 months in the present study appears more potent than the boost reported in macaques at 2 months in a previous study (*5*). The immunologic mechanism of improved boosting at later time points remains unclear, but we speculate that efficient boosting may require B cells to revert from an activated to a resting memory phenotype, which occurred gradually between days 14 and 230 after initial immunization in the present study. We similarly observed that a second immunization with Ad26.HIV.ENVA at 6 months was more efficient than at 1 month (*31*, *32*).

Our study has several limitations. This study is limited by the small number of animals per group and the lack of challenge data. The durability of immune responses after boosting also still needs to be investigated. Moreover, it is unclear whether findings in macaques will be translatable to humans.

In conclusion, we show that a late boost with Ad26.COV2.S or Ad26.COV2.S.351 resulted in a substantial increase in humoral and cellular immune responses in rhesus macaques and that these responses were highly cross-reactive against multiple SARS-CoV-2 variants. These data contribute to our understanding of Ad26.COV2.S durability and the benefit of boosting in macaques and support current clinical recommendations for Ad26.COV2.S boosting in humans.

## MATERIALS AND METHODS

### Study design

Twenty-four outbred Indian-origin adult male and female rhesus macaques (*Macaca mulatta*) aged 4 to 22 years were randomly allocated to groups. Sample size and age criteria were determined on the basis of the results of previous nonhuman primate studies. All animals were housed at BIOQUAL Inc. Animals were immunized with 10^11^ vp (*n* = 10) or 5 × 10^10^ vp (*n* = 10) Ad26.COV2.S and were followed for either 230 or 315 days. Four unvaccinated animals were used as controls. The vaccinated animals were then boosted with 5 × 10^10^ vp Ad26.COV2.S (*n* = 10) or Ad26.COV2.S.351 (*n* = 10). Half of the animals in each original dose group were immunized by each booster vaccine. All immunologic studies were performed blinded. No data points were omitted from analysis. Animal studies were conducted in compliance with all relevant local, state, and federal regulations and were approved by the BIOQUAL Institutional Animal Care and Use Committee.

### Pseudovirus-based virus neutralization assay

The SARS-CoV-2 pseudoviruses expressing a luciferase reporter gene were generated as we have previously described. In brief, the packaging plasmid psPAX2 (AIDS Resource and Reagent Program), luciferase reporter plasmid pLenti-CMV Puro-Luc (Addgene), and spike protein expressing pcDNA3.1–SARS-CoV-2 SΔCT of variants were cotransfected into human embryonic kidney (HEK) 293T cells by Lipofectamine 2000 (Thermo Fisher Scientific). Pseudoviruses of SARS-CoV-2 variants were generated using the spike protein from the WA1/2020 strain [Wuhan/WIV04/2019, Global Initiative On Sharing All Influenza Data (GISAID) accession ID: EPI_ISL_402124], B.1.1.7 variant (GISAID accession ID: EPI_ISL_601443), B.1.351 variant (GISAID accession ID: EPI_ISL_712096), P.1 variant (GISAID accession ID: EPI_ISL_792683), B.1.617.1 variant (GenBank accession ID: QTS25314.1), B.1.617.2 variant (GenBank accession ID: QTW89558.1), or B.1.1.529 variant (GISAID accession ID: EPI_ISL_7358094.2). The supernatants containing the pseudotype viruses were collected 48 hours after transfection, which were purified by centrifugation and filtration with a 0.45-μm filter. To determine the neutralization activity of the plasma or serum samples from participants, HEK293T cells expressing human angiotensin-converting enzyme 2 (HEK293T-hACE2 cells) were seeded in 96-well tissue culture plates at a density of 1.75 × 10^4^ cells per well overnight. Threefold serial dilutions of heat-inactivated serum or plasma samples were prepared and mixed with 50 μl of pseudovirus. The mixture was incubated at 37°C for 1 hour before adding to HEK293T-hACE2 cells. Forty-eight hours after infection, cells were lysed in Steady-Glo Luciferase Assay (Promega) according to the manufacturer’s instructions. SARS-CoV-2 neutralization titers were defined as the sample dilution at which a 50% reduction in relative light unit (RLU) was observed relative to the average of the virus control wells.

### Enzyme-linked immunosorbent assay

WA1/2020, B.1.1.7, B.1.351, B.1.617.1, B.1.617.2, and B.1.1.529 RBD-specific binding antibodies were assessed by ELISA essentially as described previously (*4*, *19*, *21*). In brief, 96-well plates were coated with RBD protein (1 μg/ml) in 1× Dulbecco’s phosphate-buffered saline (DPBS) and incubated at 4°C overnight. After incubation, plates were washed once with wash buffer (0.05% Tween 20 in 1× DPBS) and blocked with 350 μl of casein block per well for 2 to 3 hours at room temperature. After incubation, block solution was discarded, and plates were blotted dry. Serial dilutions of heat-inactivated serum diluted in casein block were added to wells, and plates were incubated for 1 hour at room temperature before three further washes and a 1-hour incubation with a dilution of anti-macaque IgG (1 μg/ml) conjugated to horseradish peroxidase (Nonhuman Primate Reagent Resource) at room temperature in the dark. Plates were then washed three times, and 100 μl of SeraCare KPL 3,3′,5,5′-tetramethylbenzidine (TMB) SureBlue Start solution was added to each well; plate development was halted by the addition of 100 μl of SeraCare KPL TMB Stop solution per well. The absorbance at 450 nm was recorded using a VersaMax microplate reader. For each sample, the ELISA end-point titer was calculated in GraphPad Prism software using a four-parameter logistic curve fit to calculate the reciprocal serum dilution that yields an absorbance value of 0.2 at 450 nm. Log_10_ end-point titers are reported.

### Electrochemiluminescence assay

ECLA plates [Meso Scale Discovery (MSD) SARS-CoV-2 IgG, catalog no. N05CA-1; panels 11 and 13] were designed and produced with up to 10 antigen spots in each well, and assays were performed essentially as described previously (*19*). The antigens included were WA1/2020 (ancestral), B.1.1.7 (alpha), B.1.351 (beta), P.1 (gamma), B.1.617.1 (kappa), and B.1.617.2 (delta) spike protein and RBD. The plates were blocked with 50 μl of blocker A (1% bovine serum albumin in Milli-Q water) solution for at least 30 min at room temperature with shaking at 700 rpm using a digital microplate shaker. During blocking, the serum was diluted 1:5000 in Diluent 100. The plates were then washed three times with 150 μl of the MSD Kit wash buffer and blotted dry. The diluted samples (50 μl) were added in duplicate to the plates and set to shake at 700 rpm at room temperature for at least 2 hours. The plates were again washed three times. Then, 50 μl of SULFO-tagged anti-human IgG detection antibody diluted to 1× in Diluent 100 was added to each well and incubated with shaking at 700 rpm at room temperature for at least 1 hour. Plates were then washed three times, and 150 μl of MSD GOLD Read buffer B was added to each well. The plates were read immediately after on a MESO QuickPlex SQ 120 machine. MSD titers for each sample were reported as RLU, which were calculated as sample RLU minus blank RLU for each spot for each sample. The limit of detection was defined as 1000 RLU for each assay.

### IFN-γ ELISPOT assay

ELISPOT assays were performed using PBMCs essentially as described previously (*4*, *19*, *21*). Peptide pools consisted of 15–amino acid peptides overlapping by 11 amino acids spanning the SARS-CoV-2 spike protein from the WA1/2020 strain or variant strains. ELISPOT plates were coated with mouse anti-human IFN-γ monoclonal antibody from BD Pharmigen at 5 μg per well and incubated overnight at 4°C. Plates were washed with DPBS wash buffer (DPBS with 0.25% Tween 20) and blocked with R10 media [RPMI-1640 with 10% heat-inactivated fetal bovine serum (FBS) with 1% of 100× penicillin-streptomycin] for 1 to 4 hours at 37°C. SARS-CoV-2 peptides (21st Century Biochemicals; the variants’ peptides contain the wild-type backbone) were prepared and plated at a concentration of 1 μg per well, and 200,000 cells per well were added to the plate. The peptides and cells were incubated for 18 to 24 hours at 37°C. Positive control wells were cells with phytohemagglutinin, and negative control wells were cells with media alone. All steps after this incubation were performed at room temperature. The plates were washed with ELISPOT wash buffer (11% 10× DPBS and 0.3% Tween 20 in 1 liter of Milli-Q water) and incubated for 2 hours with rabbit polyclonal anti-human IFN-γ biotin from U-CyTech (1 μg/ml). The plates were washed a second time and incubated for 2 hours with streptavidin-alkaline phosphatase from SouthernBiotech (2 μg/ml). The final wash was followed by the addition of nitro-blue tetrazolium chloride/5-bromo-4-chloro-3′-indolyphosphate p-toluidine salt (NBT/BCIP chromogen) substrate solution for 7 min. The chromogen was discarded, and the plates were washed with water and dried in a dim place for 24 hours. Plates were scanned and counted on a Cellular Technologies Limited ImmunoSpot analyzer.

### Systems serology

ADCP and ADCD assays were performed essentially as described (*10*, *27*). SARS-CoV-2 spike protein and RBD were biotinylated (Thermo Fisher Scientific) and coupled to 1-μm yellow (ADCP) and red (ADCD) fluorescent beads for 2 hours at 37°C. Excess antigen was removed by washing twice with 0.1% bovine serum albumin in PBS. Next, 1.82 × 10^8^ antigen-coated beads were added to each well of a 96-well plate and incubated with diluted samples (ADCP, 1:100; ADCD, 1:10) at 37°C for 2 hours to facilitate immune complex formation. After the incubation, complexed beads were washed, and for ADCP assays, 2.5 × 10^4^ THP-1 cells (American Type Culture Collection) were added per well and incubated for 16 hours at 37°C. For ADCD assays, lyophilized guinea pig complement was reconstituted according to the manufacturer’s instructions (Cedarlane) with water, and 4 μl per well was added in gelatin veronal buffer (GVB) containing Mg^2+^ and Ca^2+^ (GVB++, Boston BioProducts) to the immune complexes for 20 min at 37°C. After washing twice with 15 mM EDTA in PBS, immune complexes were stained with a fluorescein-conjugated goat IgG fraction to guinea pig complement C3 (MpBio). After incubation with THP-1 cells or staining of cells for ADCD, cell samples were fixed with 4% paraformaldehyde, and sample acquisition was performed via flow cytometry (Intellicyt, iQue Screener plus) using a robot arm (PAA). All events were gated on single cells and bead-positive events. For ADCP assays, a phagocytosis score was calculated as the percentage of bead-positive cells × GMFI/1000, in which GMFI denotes geometric mean fluorescence intensity. For ADCD assays, the median of C3-positive events is reported. All samples were run in duplicate on separate days.

### B cell immunophenotyping

Fresh PBMCs were stained with aqua LIVE/DEAD dye for 20 min and washed with 2% FBS/DPBS buffer, and cells were suspended in 2% FBS/DPBS buffer with Fc block (BD Biosciences) for 10 min. After blocking, samples were stained with monoclonal antibodies against CD45 [1:300; clone D058-1283, brilliant ultraviolet (BUV) 805], CD3 (1:30; clone SP34.2, allophycocyanin (APC)–Cy7), CD7 (1:30; clone M-T701, Alexa Fluor 700), CD123 (1:30; clone 6H6, Alexa Fluor 700), CD11c (1:30; clone 3.9, Alexa Fluor 700), CD19 [1:20; clone J3-119, phycoerythrin (PE)], CD20 (1:100; clone 2H7, PE-Cy5), IgA (1:90; goat polyclonal antibodies, APC), IgG (1:70; clone G18-145, BUV737), IgM (1:70; clone G20-127, BUV395), CD80 [1:40; clone L307.4, brilliant violet (BV) 786], CD95 (1:90; clone DX2, BV711), CD27 (1:170; clone M-T271, BUV563), CD21 (1:170; clone B-ly4, BV605), CD14 (1:70; clone M5E2, BV570), CD138 (1:30; clone DL-101, PE-CF594), and CD31 (1:30; clone WM59, BV785). Samples were also stained with SARS-CoV-2 antigens, including biotinylated SARS-CoV-2 (WA1/2020) RBD proteins (12.5 μg/ml; Sino Biological), full-length SARS-CoV-2 (WA1/2020) spike proteins (12.5 μg/ml; Sino Biological) labeled with fluorescein isothiocyanate, SARS-CoV-2 (B.1.351) RBD proteins (12.5 μg/ml; Sino Biological) labeled with APC, and DyLight 405. Staining was done at 4°C for 30 min. After staining, cells were washed twice with 2% FBS/DPBS buffer, followed by incubation with BV650 streptavidin (BD Pharmingen) for 10 min, and then washed twice with 2% FBS/DPBS buffer. For intracellular staining, cells were permeabilized using Caltag FIX & PERM (Thermo Fisher Scientific) and then stained with monoclonal antibodies against Ki67 (1:40; clone B56, peridinin chlorophyll protein–Cy5.5) and IRF4 (1:120; clone 3E4, PE-Cy7). After staining, cells were washed and fixed with 2% paraformaldehyde. All data were acquired on a BD FACSymphony flow cytometer. Subsequent analyses were performed using FlowJo software (BD Bioscience, v.9.9.6). For analyses, in singlet gate, dead cells were excluded by aqua dye, and CD45 was used as a positive inclusion gate for all leukocytes. Within class-switched B cell populations, gated as CD20^+^IgG^+^IgM^−^CD3^−^CD14^−^CD11c^−^CD123^−^CD7^−^, SARS-CoV-2 WA1/2020 RBD-specific B cells were identified as double positive for SARS-CoV-2 (WA1/2020) RBD and spike proteins, and SARS-CoV-2 (B.1.351) RBD-specific B cells were identified as double positive for SARS-CoV-2 (B.1.351) RBD proteins labeled with different fluorescent probes. The SARS-CoV-2–specific B cells were further distinguished according to CD21 and CD27 phenotype distribution as reported previously (*6*, *33*): activated memory B cells (CD21^−^CD27^+^) and resting memory B cells (CD21^+^CD27^+^). Within antibody-secreting PCs gated as CD20^−^CD19^+^CD138^+^CD31^+^IgM^−^IgG^+^ (*25*), SARS-CoV-2 RBD-specific PCs were identified as double positive for SARS-CoV-2 (WA1/2020) RBD and spike proteins.

### RNA sequencing

RNA was isolated from blood samples stored in PAXgene tubes at the Yerkes National Primate Center for library preparation (www.yerkes.emory.edu/nhp_genomics_core/). RNA quality was assessed using an Agilent 4200 TapeStation and concentration using the Qubit RNA HS assay (Thermo Fisher Scientific). Globin transcripts in the blood RNA were blocked with the FastSelect Globin Reagent (QIAGEN) before library preparation. Libraries were prepared using the Clontech SMART-Seq v4 Ultra Low Input RNA Kit (Takara Bio) in combination with the NexteraXT DNA Library Preparation Kit to append dual-indexed adapter sequences (Illumina). Libraries were validated by capillary electrophoresis on an Agilent 4200 TapeStation, pooled at equimolar concentrations, and sequenced on an Illumina NovaSeq 6000 at 100SR, yielding 25 to 30 million reads per sample.

Raw reads were examined for quality issues using FastQC (www.bioinformatics.babraham.ac.uk/projects/fastqc/) to ensure that library generation and sequencing are suitable for further analysis. Reads were aligned using STAR v2.7.3. Reads were aligned to *M. mulatta* genome. We used the macaque genome reference *M. mulatta*– MacaM (www.unmc.edu/rhesusgenechip/index.htm). DESeq2 was used to generate the normalized read count table based on their estimateSizeFactors() function with default parameters by calculating a pseudoreference sample of the geometric means for each gene across all samples and then using the “median ratio” of each sample to the pseudoreference as the sizeFactor for that sample. Differential expression at the gene level was performed by DESeq2 implemented in the DESeq2 R package using counts per gene generated by a custom script that pulls out the library prep abundance estimation column into files and read those files into DESeq2 with the DESeqDataSetFromHTSeqCount() function. A corrected *P* value cutoff of 0.05 was used to assess genes that were significantly up-regulated or down-regulated at days 1, 7, 14, and 28 compared to baseline using the Benjamini-Hochberg (BH) method. Raw fastq files were uploaded to the National Center for Biotechnology Information (NCBI) Gene Expression Omnibus (GEO) database under identifier GSE193264.

### Pathway enrichment analysis

GSEA and a compendium of databases of biological and immunological pathways were used to test the longitudinal enrichment of pathways on days 1, 7, 14, and 28 after vaccination compared with baseline on day 0. Genes were preranked by fold change from the highest to the lowest, and GSEA was used to assess the enrichment of selected gene sets. Cytokines signaling, immune cell signatures, and molecular pathways were compiled from the MSigDB Hallmark, C2, C7, and C3 gene sets (www.gsea-msigdb.org/gsea/msigdb/collections.jsp), and the blood transcriptional modules (*34*). The GSEA Java desktop program was downloaded from the Broad Institute (www.broadinstitute.org/gsea/index.jsp) and used with GSEA preranked module parameters (number of permutations: 1000; enrichment statistic: weighted; 10 ≤ gene set size ≤ 5000). Sample-level enrichment analysis (*35*) was used to investigate the enrichment of pathways in each animal. In brief, the expression of all the genes in a specific pathway was averaged across samples and compared to the average expression of 1000 randomly generated gene sets of the same size. The resulting *Z* score was then used to reflect the overall perturbation of each pathway in each individual sample. Pathways’ sample-level enrichment scores were correlated with binding and neutralizing titers and B cell responses elicited by Ad26.COV2.S at the indicated days after vaccination using the Spearman correlation method.

### Pharmacokinetics

To estimate antibody decay rate, the median values for 1 × 10^11^ and 5 × 10^10^ dose groups were plotted as a function of time after vaccination, and the data were fitted to a biphasic decaying exponential model using the curve fitting tool in MATLAB (version R_2021a). The half-life of the fast and slow phases of each decay model is indicated on the graphs (fig. S1). The day on which the decay function transitions from fast to slow decay is indicated in the blue dashed line for the 1 × 10^11^ dose and in the orange dashed line for the 5 × 10^10^ dose. The biphasic exponential decay is mathematically represented by

$$V(t)={\mathrm{Ae}}^{\partial t}+Be^{\omega t}$$ (1)

where *V*(*t*) is the antibody titer as a function of time; *A + B* = *V*_0_, where *V*_0_ the initial antibody titer at time *t*_0_ (*t* = 0); and ∂ and ω < 0 are the decay rates of antibody titers.

To model each curve, time *t*_0_ was taken to be the day after vaccination when peak antibody responses were reached, and the remaining time points were adjusted accordingly. For example, for the 1 × 10^11^ vp dose ELISA titers, peak antibody responses were observed on day 28 after vaccination. To model the antibody titer decay, day 28 was treated as *t* = 0 (*t*_0_). This is due to the nature of the decaying exponential function: since *t* cannot take on negative values for our model, *t =* 0 is when the exponential decay reaches its maximum value. Therefore, the day that corresponds to peak antibody titers was treated as *t*_0_. The data were then plotted as a function of days after vaccination.

As per Eq. 1, the exponential function has two terms. The first term corresponds to the “fast” term, as this is when we observe a sharp decrease in antibody titers. The second term corresponds to the “slow” term, where the rate of antibody decay is lower. However, there is a given time denoted by a blue and orange dashed line in fig. S1, where we observe a transition from fast decay to low decay, such that

$$V(t^{*})=Ae^{\partial t^{*}}=Be^{\omega t^{*}}$$ (2)

Furthermore, we characterized the antibody half-life for the fast and slow phases, defined as the time it takes for antibody titers to decrease by half of their initial value. To find the half-life, we equate Eq. 1 to $\frac{V_{0}}{2}$. For the fast phase, this is mathematically represented as

$$\frac{V_{0}}{2}={\mathrm{Ae}}^{\partial t}+Be^{\omega t}$$ (3)

As such, solving Eq. 3 for *t* yields the half-life for the fast phase, which is indicated in the blue in fig. S1 as *t*_1/2_. Similarly, to determine the half-life for the slow phase, we can use

$$\frac{V_{0}^{*}}{2}={\mathrm{Ae}}^{\partial t}+Be^{\omega t}$$ (4)

In Eq. 4, $V_{0}^{*}$ is the amount of antibody titer at time t^*^ (given by solving Eq. 2), such that $V_{0}^{*}=V(t^{*})$. Hence, solving for *t* in Eq. 4 gives the slow-phase half-life, which is indicated in orange in fig. S1 as *t*_1/2_.

### Statistical analysis

All raw, individual-level data are presented in data files S1 to S6. Comparisons of immunologic data were performed using GraphPad Prism 8.4.2 (GraphPad Prism software). Comparison of data between groups was performed using two-sided Mann-Whitney tests or Wilcoxon signed-rank tests (for matched pairs). *P* values of less than 0.05 were considered significant. For differential expression gene analyses, *P* values were corrected for multiple testing using the BH method and a cutoff of 0.05. Pathway enrichment analysis was performed using R and was assessed using an FDR cutoff of 5%. Correlation analyses were performed using the cor.test R package, and statistical significance was assessed using two-sided Spearman rank correlation tests.

## Supplementary Material

20220222-1

## Supplementary Materials

### Other Supplementary Material for this manuscript includes the following:

Data files S1 to S6

MDAR Reproducibility Checklist

## Notes

View/request a protocol for this paper from *Bio-protocol*.
