# Supplementary material for: A homologous or variant booster vaccine after Ad26.COV2.S immunization enhances SARS-CoV-2–specific immune responses in rhesus macaques
Source: Sci Transl Med. 2022 Mar 30;14(638):eabm4996. doi: 10.1126/scitranslmed.abm4996 (PMC9802654; doi:10.1126/scitranslmed.abm4996)
Supplement: 20220222-1 [file scitranslmed.abm4996.v1.pdf]

Cite as: X. He *et al.*, *Sci. Transl. Med.* 10.1126/scitranslmed.abm4996 (2022).

CORONAVIRUS

A homologous or variant booster vaccine after Ad26.COV2.S immunization enhances SARS-CoV-2-specific immune responses in rhesus macaques

Xuan He[1†], Malika Aid[1†], Abishek Chandrashekar[1†], Jingyou Yu[1†], Katherine McMahan[1†], Frank Wegmann[2†], Catherine Jacob-Dolan[1,3†], Jenny S. Maron[3,4], Caroline Atyeo[3,4], Huahua Wan[1], Daniel Sellers[1], Jinyan Liu[1], Michelle Lifton[1], Sarah Gardner[1], Esther A. Bondzie[1], Julia Barrett[1], Kunza Ahmad[1], Tochi Anioke[1], Jake Yalley-Ogunro[5], Jeanne Muench[5], Adrienne Goode[5], Hanne Andersen[5], Mark G. Lewis[5], Galit Alter[3,4], Hanneke Schuitemaker[2,6], Roland Zahn[2], and Dan H. Barouch[1,3,4,*]

[1]Center for Virology and Vaccine Research, Beth Israel Deaconess Medical Center, Harvard Medical School, Boston, MA 02215, USA; [2]Janssen Vaccines & Prevention, Leiden, Netherlands; [3]Harvard Medical School, Boston, MA 02115, USA; [4]Ragon Institute of MGH, MIT and Harvard, Cambridge, MA, USA; [5]Bioqual, Rockville, MD 20852, USA; [6]Amsterdam University Medical Center, Amsterdam, Netherlands

†These authors contributed equally to this work

***Corresponding author. Email: dbarouch@bidmc.harvard.edu**

Ad26.COV2.S has demonstrated durability and clinical efficacy against symptomatic COVID-19 in humans. In this study, we report the correlates of durability of humoral and cellular immune responses in 20 rhesus macaques immunized with single-shot Ad26.COV2.S and the immunogenicity of a booster shot at 8 to 10 months following the initial immunization. Ad26.COV2.S elicited durable binding and neutralizing antibodies as well as memory B cells and long-lived bone marrow plasma cells. Innate immune responses and bone marrow plasma cell responses correlated with durable antibody responses. Following Ad26.COV2.S boost immunization, binding and neutralizing antibody responses against multiple SARS-CoV-2 variants increased 31- to 69-fold and 23- to 43-fold, respectively, compared with pre-boost concentrations. Antigen-specific B cell and T cell responses also increased substantially following the boost immunization. Boosting with a modified Ad26.COV2.S.351 vaccine expressing the SARS-CoV-2 spike protein from the beta variant led to largely comparable responses with slightly higher beta- and omicron-specific humoral immune responses. These data demonstrate that a late boost with Ad26.COV2.S or Ad26.COV2.S.351 resulted in a dramatic increase in humoral and cellular immune responses that were highly cross-reactive across multiple SARS-CoV-2 variants in rhesus macaques.

INTRODUCTION

The Ad26.COV2.S vaccine is a replication-incompetent adenovirus (Ad) 26 vector (*1*) expressing the stabilized severe acute respiratory syndrome coronavirus 2 (SARS-CoV-2) spike protein (*2*) from the Wuhan 2019 strain, which is identical to the spike protein from the WA1/2020 strain. Immunogenicity and protective efficacy of the single-shot Ad26.COV2.S vaccine has been demonstrated in hamsters and rhesus macaques (*3*–*6*) as well as in humans (*4*, *7*–*11*). Recent data has also shown durability of immune responses induced by single-shot Ad26.COV2.S in humans for at least 8 months (*12*). However, waning immunity has also been reported for coronavirus disease 2019 (COVID-19) vaccines (*13*), and recent reports of breakthrough infections with the SARS-CoV-2 variants, including delta (B.1.617.2) and omicron (B.1.1.529), in fully vaccinated individuals (*14*–*17*) have subsequently highlighted the need for boost immunizations for all the currently approved vaccines, including Ad26.COV2.S.

Moreover, immune imprinting has been observed following both COVID-19 infection (*18*) and immunizations (*19*). By immune imprinting, the initial COVID-19 vaccination might shape the subsequent immune responses against variants of concern after homologous or heterologous boosts. In this study, we evaluated the correlates of durability of single-shot Ad26.COV2.S vaccination in rhesus macaques and the immunogenicity of a late boost at 8-10 months with Ad26.COV2.S or Ad26.COV2.S.351, which expresses the stabilized SARS-CoV-2 Spike protein from the B.1.351 (beta) variant.

RESULTS

Durability of Immune Responses Following Single-Shot Ad26.COV2.S Vaccination

We first assessed the durability of humoral and cellular immune responses following single-shot Ad26.COV2.S vaccination. Twenty rhesus macaques were immunized by the intramuscular route with 10^{11} viral particles (vp) (n=10) or

$5x10^{10}$ vp (n=10) of Ad26.COV2.S and were followed for either 230 or 315 days, which reflected a staggered start for half of the animals in each dose group (**Fig. 1**). An additional 4 animals were included as sham controls. Receptor binding domain (RBD)-specific binding antibody responses were assessed by enzyme-linked immunosorbent assay (ELISA, **Fig. 2A**) (*4*, *20*, *21*). WA1/2020 RBD-specific ELISA responses peaked on days 28 to 56 at median titers of 7,413 (interquartile range [IQR]: 3,802 to 14,835) and 6,478 (IQR: 4,173 to 10,696) with the 10^{11} vp and $5x10^{10}$ vp doses, respectively, and then showed a biphasic decay with half-lives of 38 days and 139 to 254 days, respectively (**fig. S1**). All animals showed binding antibody responses for the duration of follow-up with no difference between the two doses tested. Neutralizing antibody (NAb) responses against SARS-CoV-2 WA1/2020 were assessed by a luciferase-based pseudovirus NAb (psVNA) assay (*4*, *20–22*) and showed similar kinetics to the binding antibody responses (**Fig. 2B**). NAb responses peaked on days 28 to 56 at median titers of 763 (IQR: 534 to 1,179) and 649 (IQR: 408 to 904) with the 10^{11} vp and $5x10^{10}$ vp doses, respectively, and then showed a biphasic decay with half-lives of 30 to 44 days and 184 to 247 days (**fig. S1**). Seventeen of 20 animals showed NAb responses by day 230 to 315. Similar magnitudes and kinetics of antibody responses were observed for the two vaccine doses tested.

Spike (S) protein-specific cellular immune responses were assessed by pooled peptide enzyme-linked immune absorbent spot (ELISPOT) assays using overlapping 15 amino acid peptides in peripheral blood mononuclear cells (PBMCs). Interferon (IFN)-γ ELISPOT responses to SARS-CoV-2 WA1/2020 spike protein peaked on day 14 and then declined gradually over 230 days (**fig. S2**). ELISPOT responses were similar for the two vaccine doses tested.

Antigen-Specific B Cell and Plasma Cell Responses

Long-lived antigen-specific IgG^+ memory B cells are important for generating anamnestic responses following antigen re-exposure. WA1/2020 RBD -specific IgG^+ memory B cells were assessed longitudinally in PBMCs by multiparameter flow cytometry (*6*). Ad26.COV2.S elicited RBD-specific memory B cells in all macaques by day 14 to 28 followed by a gradual decline, and specific B cell responses remained detectable in 17 of 20 animals by day 230 to 315 with no differences between the two doses tested (**Fig. 3A**). RBD-specific memory B cells initially exhibited an activated memory phenotype (AM; $CD21^-CD27^+$), which gradually transitioned into a resting memory phenotype (RM; $CD21^+CD27^+$) between days 14 and 230 following immunization (**Fig. 3B; fig. S3**). The frequencies of RBD-specific memory B cells on day 28 correlated with binding and NAb titers on day 28 (P<0.0001, R=0.7614 and P<0.0001, R=0.7306, respectively, two-sided Spearman rank-correlation tests; **fig. S4**).

Long-lived plasma cells (PCs) in bone marrow are important for maintaining durable circulating antibodies (*23–25*). Bone marrow aspirates were collected from 10 vaccinated macaques on day 315 (half from each dose group) and from 4 unvaccinated sham control macaques. $CD138^+CD31^+$ PCs have been reported to be highly enriched for IgG-secreting B cells (*26*). We observed high expression of IgG and CD95 and low expression of Ki67 on $CD138^+CD31^+$ PCs, as expected (**fig. S5**). On day 315 following vaccination, bone marrow WA1/2020 RBD-specific PCs were detected in the 7 out of 10 vaccinated macaques (**Fig. 4, A and B**). Bone marrow RBD-specific PCs correlated with NAb titers on day 252 (P=0.0091, R=0.7100, two-sided Spearman rank-correlation tests) and with binding and NAb titers on day 315 (P=0.0436, R=0.5530 and P=0.0310, R=0.5881, respectively, two-sided Spearman rank-correlation tests; **Fig. 4C**).

Transcriptomic Signatures of Durability

To define transcriptomic signatures following Ad26.COV2.S vaccination and correlations with durable B cell and antibody responses, we performed bulk RNA sequencing of PBMCs on days 0, 1, 7, 14, and 28 in 10 animals that had sufficient cells following Ad26.COV2.S vaccination (half from each dose group). Gene set enrichment analyses (GSEA) revealed up-regulation of B cell pathways (**Fig. 5A**). Signatures of T follicular helper (Tfh) cells, B cells, B cell receptor (BCR) signaling, and plasma cells were up-regulated on days 7 to 28 (FDR q<0.05), and signatures of natural killer T (NKT) cells were up-regulated on days 1 to 28 (FDR q<0.05) following vaccination (**Fig. 5A**).

Innate pathways, including type I interferon responses, IFN-γ responses, RIG-1 like receptor signaling, mucosal-associated invariant T (MAIT) cell signatures, natural killer (NK) cell signatures, and B cell lymphoma 2 (BCL2) survival signaling targets, were also up-regulated (FDR q<0.05) on day 1 following vaccination and then rapidly resolved (**Fig. 5B**). These pathways correlated with RBD-specific memory B cell responses at both early (day 28) and late (day 112) time points (**Fig. 5C**). NK cell signatures on day 1 also correlated with long-term plasma cell responses (day 315) (**Fig. 5C**). In addition, type I interferon signatures correlated with binding and NAb titers at multiple time points following immunization (**Fig. 5D**). These data show that early innate responses following vaccination correlated with durable B cell, plasma cell, and antibody responses.

Immunogenicity of Ad26.COV2.S or Ad26.COV2.S.351 Boost Immunization

All 20 vaccinated macaques received a boost immunization at 8 or 10 months following the initial immunization, which reflected the staggered start of the original study. Animals received a boost with $5x10^{10}$ vp Ad26.COV2.S (n=10) or Ad26.COV2.S.351 (n=10), which expresses the stabilized spike protein from the SARS-CoV-2 B.1.351 (beta) variant. Of note, the $5x10^{10}$ vp dose is the clinically approved dose for

Ad26.COV2.S for both priming and boosting. Prior to the boost, binding and NAb responses against SARS-CoV-2 variants were observed with median ELISA titers of 278 (IQR: 135 to 776) to 812 (IQR: 553 to 1682), and median NAb titers of 37 (IQR: 31 to 41) to 67 (IQR: 48 to 119) (**Fig. 6, A and B**). By day 14 following the Ad26.COV2.S boost immunization, RBD-specific binding antibody responses increased 31- to 69-fold compared with pre-boost values against the ancestral (WA1/2020), alpha (B.1.1.7), beta (B.1.351), kappa (B.1.617.1), and delta (B.1.617.2) SARS-CoV-2 variants (P<0.0001 for all, two-sided Mann-Whitney tests; **Fig. 6A**). By day 14, NAb responses increased 23- to 43-fold compared with pre-boost values against the ancestral, alpha, beta, gamma (P.1), kappa, and delta SARS-CoV-2 variants (P<0.0001 for all, two-sided Mann-Whitney tests; **Fig. 6B**). Twenty-eight days following the boost immunization, NAb responses to the beta and delta variants were only 1.9- and 1.7-fold lower than the WA1/2020 response, respectively, following the boost immunization (P>0.05, not significant for both, two-sided Mann-Whitney tests). At day 28 post-boost immunization, binding and NAb responses against omicron were 7.7- and 3.4-fold lower than WA1/2020 responses, respectively, following boosting (P<0.0001 for both, two-sided Mann-Whitney tests). WA1/2020-specific ELISA and NAb responses following the boost were 6.8- and 2.8-fold greater, respectively, than peak responses on days 28 to 56 following the initial single-shot Ad26.COV2.S immunization (P<0.0001 and P<0.0001, respectively, two-sided Mann-Whitney tests; **Fig. 6C**), suggesting that the boost immunization led to robust anamnestic antibody responses. Boosting with Ad26.COV2.S.351 resulted in largely comparable humoral immune responses, with 1.9- and 2.8-fold higher beta-specific NAb responses on days 14 and 28, respectively (P<0.05 for both, two-sided Mann-Whitney tests) and with a 1.8-fold higher omicron-specific binding antibody response on day 28 (P < 0.05, two-sided Mann-Whitney tests) compared with Ad26.COV2.S boosting (**Fig. 6 A and B**).

An electrochemiluminescence assay (ECLA) (*27*) was also utilized to evaluate RBD- and S-specific binding antibody responses to multiple SARS-CoV-2 variants on day 28 and day 230/315 following initial immunization and on day 14 following the boost immunization and similarly demonstrated substantial boosting to all antigens tested (P<0.0001 for all, two-sided Mann-Whitney tests; **fig. S6**). Following the boost immunization, robust antibody-dependent cellular phagocytosis (ADCP) and antibody-dependent complement deposition (ADCD) (*10*, *28*) were also observed against multiple variants (**fig. S7**).

Boosting with Ad26.COV2.S or Ad26.COV2.S.351 led to rapid and robust B cell memory recall responses with a 4- to 10-fold increase in RBD-specific B cells at day 14 relative to day 0 (P<0.01 for both, two-sided Mann-Whitney tests; **Fig. 6D**), which showed an activated memory B cell phenotype (**fig. S8**). Boost immunization also induced a 4- to 23-fold increase in T cell responses by IFN-γ ELISPOT assays at day 14 (P<0.001 for both, two-sided Mann-Whitney tests; **Fig. 6E**). Humoral and cellular immune responses were largely comparable across all the variants studied following the boost immunization, suggesting substantial cross-reactivity against SARS-CoV-2 variants.

DISCUSSION

The single-shot Ad26.COV2.S vaccine has demonstrated protective efficacy in humans in the United States, Latin America, and South Africa in the phase 3 ENSEMBLE study, including against the SARS-CoV-2 beta variant (*9*), as well as real-world effectiveness against severe disease from the SARS-CoV-2 delta and omicron variants. We recently reported durable humoral and cellular immune responses with minimal evidence of decline in humans over a period of 8 months following Ad26.COV2.S vaccination (*12*). However, increased breakthrough infections with the highly transmissible SARS-CoV-2 delta and omicron variants in fully vaccinated individuals have led to recommendations for booster shots for all the currently approved vaccines. A deeper understanding of the durability and benefit of boosting with Ad26.COV2.S is therefore needed.

In this study, we report the durability of single-shot Ad26.COV2.S vaccination in rhesus macaques and the immunogenicity of a boost immunization at 8 to 10 months. Durability of single-shot Ad26.COV2.S vaccination has also been reported in humans (*12*, *29*); following Ad26.COV2.S vaccination, neutralizing antibodies were 3-fold higher in humans than reported here in macaques (*29*). We also reported a NAb threshold for protection against SARS-CoV-2 challenge in a model of short-term natural immunity in macaques (*30*). In the present study, 14 of 20 macaques showed durable NAb titers above this threshold.

Here, we showed that single-shot Ad26.COV2.S vaccination induced durable antigen-specific memory B cells and bone marrow plasma cells. The frequencies of RBD-specific plasma cells residing in bone marrow correlated with serum binding and neutralizing antibodies. In addition, we observed that early activation of interferon and inflammatory pathways correlated with durable B cell, plasma cell, and antibody responses, reflecting the impact of innate immunity on humoral immune memory.

A boost immunization with Ad26.COV2.S or Ad26.COV2.S.351 in macaques led to a substantial 1 to 2 log increase in binding and NAb titers to values that were higher than peak responses following initial immunization, as well as markedly enhanced memory B cell and T cell responses. Moreover, humoral and cellular immune responses following the boost immunization were highly cross-reactive against multiple SARS-CoV-2 variants, including the alpha, beta,

gamma, and delta variants, with only minimal evidence for immune imprinting. Humoral responses against the omicron variant were lower, consistent with published data (*14–16*). Boosting with Ad26.COV2.S.351 led to modestly higher beta-specific and omicron-specific humoral immune responses. Moreover, the efficiency of the observed boosting suggests that anti-vector immunity following initial immunization (*11*) does not preclude a second immunization with homologous Ad26.COV2.S or heterologous Ad26.COV2.S.351. Although the mechanisms of protection against SARS-CoV-2 in humans remain to be determined, we speculate that the substantially enhanced immune responses following boost immunization will lead to improved protective efficacy. Of note, we previously demonstrated that both antibodies and T cells in convalescent rhesus macaques contributed to protection against SARS-CoV-2 challenge (*30*).

Boosting humans at 6 months with Ad26.COV2.S has been shown to result in a 9-fold increase in antibody titers (*31*), whereas boosting humans at 2 months led to a 3- to 4-fold increase in antibody titers (*32*). Similarly, the boost observed in macaques at 8 to 10 months in the present study appears more potent than the boost reported in macaques at 2 months in a previous study (*5*). The immunologic mechanism of improved boosting at later time points remains unclear, but we speculate that efficient boosting may require B cells to revert from an activated to a resting memory phenotype, which occurred gradually between days 14 and 230 following initial immunization in the present study. We similarly observed that a second immunization with Ad26.HIV.ENVA at 6 months was more efficient than at 1 month (*33*, *34*).

Our study has several limitations. This study is limited by the small number of animals per group and the lack of challenge data. The durability of immune responses following boosting also still needs to be investigated. Moreover, it is unclear whether findings in macaques will be translatable to humans.

In conclusion, we show that a late boost with Ad26.COV2.S or Ad26.COV2.S.351 resulted in a substantial increase in humoral and cellular immune responses in rhesus macaques, and that these responses were highly cross-reactive against multiple SARS-CoV-2 variants. These data contribute to our understanding of Ad26.COV2.S durability and the benefit of boosting in macaques and support current clinical recommendations for Ad26.COV2.S boosting in humans.

MATERIALS AND METHODS

Study design

Twenty-four outbred Indian-origin adult male and female rhesus macaques (*Macaca mulatta*) ages 4 to 22 years old were randomly allocated to groups. Sample size and age criteria were determined based on the results of previous non-human primate studies. All animals were housed at Bioqual, Inc. Animals were immunized with 10^{11} vp (n=10) or $5x10^{10}$ vp (n=10) Ad26.COV2.S and were followed for either 230 or 315 days. Four unvaccinated animals were utilized as controls. The vaccinated animals were then boosted with $5x10^{10}$ vp Ad26.COV2.S (n=10) or Ad26.COV2.S.351 (n=10). Half of the animals in each original dose group were immunized by each booster vaccine. All immunologic studies were performed blinded. No data points were omitted from analysis. Animal studies were conducted in compliance with all relevant local, state, and federal regulations and were approved by the Bioqual Institutional Animal Care and Use Committee (IACUC).

Pseudovirus-based virus neutralization assay

The SARS-CoV-2 pseudoviruses expressing a luciferase reporter gene were generated as we have previously described. Briefly, the packaging plasmid psPAX2 (AIDS Resource and Reagent Program), luciferase reporter plasmid pLenti-CMV Puro-Luc (Addgene), and spike protein expressing pcDNA3.1-SARS CoV-2 S∆CT of variants were co-transfected into HEK293T cells by lipofectamine 2000 (Thermo Fisher Scientific). Pseudoviruses of SARS-CoV-2 variants were generated using the spike protein from the WA1/2020 strain (Wuhan/WIV04/2019, Global Initiative On Sharing All Influenza Data [GISAID] accession ID: EPI_ISL_402124), B.1.1.7 variant (GISAID accession ID: EPI_ISL_601443), B.1.351 variant (GISAID accession ID: EPI_ISL_712096), P.1 variant (GISAID accession ID: EPI_ISL_792683), B.1.617.1 variant (GenBank accession ID: QTS25314.1), B.1.617.2 variant (GenBank Accession ID: QTW89558.1), or B.1.1.529 variant (GISAID accession ID: EPI_ISL_7358094.2). The supernatants containing the pseudotype viruses were collected 48 hours post-transfection, which were purified by centrifugation and filtration with 0.45 µm filter. To determine the neutralization activity of the plasma or serum samples from participants, HEK293T cells expressing human angiotensin-converting enzyme 2 (HEK293T-hACE2 cells) were seeded in 96-well tissue culture plates at a density of 1.75×10^4 cells per well overnight. Three-fold serial dilutions of heat-inactivated serum or plasma samples were prepared and mixed with 50 µL of pseudovirus. The mixture was incubated at 37°C for 1 hour before adding to HEK293T-hACE2 cells. Forty-eight hours after infection, cells were lysed in Steady-Glo Luciferase Assay (Promega) according to the manufacturer's instructions. SARS-CoV-2 neutralization titers were defined as the sample dilution at which a 50% reduction in relative light unit (RLU) was observed relative to the average of the virus control wells.

ELISA

WA1/2020, B.1.1.7, B.1.351, B.1.617.1, B.1.617.2, or B.1.1.529 RBD-specific binding antibodies were assessed by ELISA essentially as described previously (*4*, *20*, *22*). Briefly, 96-well plates were coated with 1 µg/ml RBD protein in 1X Dulbecco's phosphate-buffered saline (DPBS) and incubated at 4°C overnight. After incubation, plates were washed once with wash

buffer (0.05% Tween 20 in 1X DPBS) and blocked with 350 μL Casein block per well for 2 to 3 hours at room temperature. After incubation, block solution was discarded, and plates were blotted dry. Serial dilutions of heat-inactivated serum diluted in casein block were added to wells, and plates were incubated for 1 hour at room temperature, prior to three further washes and a 1 hour incubation with a 1μg/ml dilution of anti-macaque IgG conjugated to horseradish peroxidase (HRP) (Nonhuman Primate Reagent Resource) at room temperature in the dark. Plates were then washed three times, and 100 μL of SeraCare KPL 3,3′,5,5′-Tetramethylbenzidine (TMB) SureBlue Start solution was added to each well; plate development was halted by the addition of 100 μL SeraCare KPL TMB Stop solution per well. The absorbance at 450nm was recorded using a VersaMax microplate reader. For each sample, the ELISA endpoint titer was calculated in GraphPad Prism software, using a four-parameter logistic curve fit to calculate the reciprocal serum dilution that yields an absorbance value of 0.2 at 450nm. Log10 endpoint titers are reported.

Electrochemiluminescence assay (ECLA)

ECLA plates (MesoScale Discovery SARS-CoV-2 IgG Cat No: N05CA-1; Panels 11 and 13) were designed and produced with up to 10 antigen spots in each well, and assays were performed essentially as described previously (*20*). The antigens included were WA1/2020 (ancestral), B.1.1.7 (alpha), B.1.351 (beta), P.1 (gamma), B.1.617.1 (kappa), and B.1.617.2 (delta) spike protein and RBD. The plates were blocked with 50 μL of Blocker A (1% bovine serum albumin in MilliQ water) solution for at least 30 min at room temperature shaking at 700 rpm with a digital microplate shaker. During blocking, the serum was diluted 1:5,000 in Diluent 100. The plates were then washed 3 times with 150 μL of the MSD kit Wash Buffer and blotted dry. Fifty μL of the diluted samples were added in duplicate to the plates and set to shake at 700 rpm at room temperature for at least 2 hours. The plates were again washed 3 times. Then, 50 μL of SULFO-Tagged anti-Human IgG detection antibody diluted to 1X in Diluent 100 was added to each well and incubated with shaking at 700 rpm at room temperature for at least 1 hour. Plates were then washed 3 times, and 150 μL of MSD GOLD Read Buffer B was added to each well. The plates were read immediately after on a MESO QuickPlex SQ 120 machine. MSD titers for each sample were reported as Relative Light Units (RLU), which were calculated as Sample RLU minus Blank RLU for each spot for each sample. The limit of detection was defined as 1000 RLU for each assay.

IFN-γ enzyme-linked immunospot (ELISPOT) assay

ELISPOT assays were performed using PBMCs essentially as described previously (*4*, *20*, *22*). Peptide pools consisted of 15 amino acid peptides overlapping by 11 amino acids spanning the SARS-CoV-2 Spike protein from the WA1/2020 strain or variant strains. ELISPOT plates were coated with mouse anti-human IFN-γ monoclonal antibody from BD Pharmigen at 5 μg/well and incubated overnight at 4°C. Plates were washed with DPBS wash buffer (DPBS with 0.25% Tween 20) and blocked with R10 media (RPMI-1640 with 10% heat-inactivated fetal bovine serum [FBS] with 1% of 100x penicillin-streptomycin) for 1 to 4 hours at 37°C. SARS-CoV-2 peptides (21st Century Biochemicals; the variants peptides contain the wild-type backbone) were prepared and plated at a concentration of 1 μg/well, and 200,000 cells per well were added to the plate. The peptides and cells were incubated for 18 to 24 hours at 37°C. Positive control wells were cells with phytohemagglutinin, and negative control wells were cells with media alone. All steps following this incubation were performed at room temperature. The plates were washed with ELISPOT wash buffer (11% 10X DPBS and 0.3% Tween 20 in 1L MilliQ water) and incubated for 2 hours with rabbit polyclonal anti-human IFN-γ Biotin from U-Cytech (1 μg/mL). The plates were washed a second time and incubated for 2 hours with streptavidin-alkaline phosphatase from Southern Biotech (2 μg/mL). The final wash was followed by the addition of nitro-blue tetrazolium chloride/5-bromo-4-chloro 3′-indolyphosphate p-toluidine salt (NBT/BCIP chromogen) substrate solution for 7 min. The chromogen was discarded, and the plates were washed with water and dried in a dim place for 24 hours. Plates were scanned and counted on a Cellular Technologies Limited Immunospot Analyzer.

Systems serology

Antibody-dependent cellular phagocytosis (ADCP) and antibody-dependent complement deposition (ADCD) assays were performed essentially as described (*10*, *28*). SARS-CoV-2 spike protein and RBD were biotinylated (Thermo Fisher Scientific) and coupled to 1 μm yellow (ADCP) and red (ADCD) fluorescent beads for 2 hours at 37°C. Excess antigen was removed by washing twice with 0.1% bovine serum albumin in phosphate-buffered saline (PBS). Next, 1.82×10^8 antigen-coated beads were added to each well of a 96-well plate and incubated with diluted samples (ADCP 1:100, ADCD 1:10) at 37°C for 2 hours to facilitate immune complex formation. After the incubation, complexed beads were washed, and, for ADCP assays, 2.5×10^4 THP-1 cells (American Type Culture Collection) were added per well and incubated for 16 hours at 37°C. For ADCD assays, lyophilized guinea pig complement was reconstituted according to manufacturer's instructions (Cedarlane) with water, and 4 μl per well were added in gelatin veronal buffer containing Mg2+ and Ca^{2+} (GVB++, Boston BioProducts) to the immune complexes for 20 min at 37°C. After washing twice with 15 mM EDTA in PBS, immune complexes were stained with a fluorescein-conjugated goat IgG fraction to guinea pig complement C3 (MpBio). After incubation with THP-1 cells or staining of cells for ADCD, cell samples were fixed with 4% paraformaldehyde, and sample

acquisition was performed via flow cytometry (Intellicyt, iQue Screener plus) using a robot arm (PAA). All events were gated on single cells and bead-positive events. For ADCP assays, a phagocytosis score was calculated as the percentage of bead positive cells × GMFI/1000, in which GMFI denotes geometric mean fluorescence intensity. For ADCD assays, the median of C3-positive events is reported. All samples were run in duplicate on separate days.

B cell immunophenotyping

Fresh PBMCs were stained with Aqua live/dead dye for 20 min, washed with 2% FBS/DPBS buffer, and cells were suspended in 2% FBS/DPBS buffer with Fc Block (BD Biosciences) for 10 min. After blocking, samples were stained with monoclonal antibodies against CD45 (clone D058-1283, brilliant ultraviolet (BUV) 805, 1:300), CD3 (clone SP34.2, allophycocyanin (APC)-Cy7, 1:30), CD7 (clone M-T701, Alexa Fluor700, 1:30), CD123 (clone 6H6, Alexa Fluor 700, 1:30), CD11c (clone 3.9, Alexa Fluor 700, 1:30), CD19 (clone J3-119, phycoerythrin (PE), 1:20), CD20 (clone 2H7, PE-Cy5, 1:100), IgA (goat polyclonal antibodies, APC, 1:90), IgG (clone G18-145, BUV737, 1:70), IgM (clone G20-127, BUV395, 1:70), CD80 (clone L307.4, brilliant violet (BV) 786, 1:40), CD95 (clone DX2, BV711, 1:90), CD27 (clone M-T271, BUV563, 1:170), CD21 (clone B-ly4, BV605, 1:170), CD14 (clone M5E2, BV570, 1:70), CD138 (clone DL-101, PE-CF594, 1:30), CD31 (clone WM59, BV785, 1:30). Samples were also stained with SARS-CoV-2 antigens, including biotinylated SARS-CoV-2 (WA1/2020) RBD proteins (Sino Biological, 12.5 μg/ml), full-length SARS-CoV-2 (WA1/2020) spike proteins (Sino Biological, 12.5 μg/ml) labeled with fluorescein isothiocyanate (FITC), SARS-CoV-2 (B.1.351) RBD proteins (Sino Biological, 12.5 μg/ml) labeled with APC and DyLight 405. Staining was done at 4°C for 30 min. After staining, cells were washed twice with 2% FBS/DPBS buffer, followed by incubation with BV650 streptavidin (BD Pharmingen) for 10 min, then washed twice with 2% FBS/DPBS buffer. For intracellular staining, cells were permeabilized using Caltag Fix & Perm (Thermo Fisher Scientific), then stained with monoclonal antibodies against Ki67 (clone B56, peridinin chlorophyll protein (PerCP)-Cy5.5, 1:40) and IRF4 (clone 3E4, PE-Cy7, 1:120). After staining, cells were washed and fixed with 2% paraformaldehyde. All data were acquired on a BD FACSymphony flow cytometer. Subsequent analyses were performed using FlowJo software (BD Bioscience, v.9.9.6). For analyses, in singlet gate, dead cells were excluded by Aqua dye, and CD45 was used as a positive inclusion gate for all leukocytes. Within class-switched B cell populations, gated as $CD20^{+}IgG^{+}IgM^{-}CD3^{-}CD14^{-}CD11c^{-}CD123^{-}CD7^{-}$, SARS-CoV-2 WA1/2020 RBD-specific B cells were identified as double-positive for SARS-CoV-2 (WA1/2020) RBD and spike proteins, and SARS-CoV-2 (B.1.351) RBD-specific B cells were identified as double-positive for SARS-CoV-2 (B.1.351) RBD proteins labeled with different fluorescent probes. The SARS-CoV-2-specific B cells were further distinguished according to CD21 and CD27 phenotype distribution as previously reported (*6*, *35*): activated memory B cells ($CD21^{-}CD27^{+}$) and resting memory B cells ($CD21^{+}CD27^{+}$). Within antibody-secreting plasma cells gated as $CD20^{-}CD19^{+}CD138^{+}CD31^{+}IgM^{-}IgG^{+}$ (*26*), SARS-CoV-2 RBD-specific plasma cells were identified as double-positive for SARS-CoV-2 (WA1/2020) RBD and spike proteins.

RNA sequencing

RNA was isolated from blood samples stored in Paxgene tubes at the Yerkes National Primate Center for library preparation (http://www.yerkes.emory.edu/nhp_genomics_core/). RNA quality was assessed using an Agilent 4200 TapeStation and concentration using the Qubit RNA HS assay (Thermo Fisher Scientific). Globin transcripts in the blood RNA were blocked with the FastSelect Globin Reagent (Qiagen) prior to library preparation. Libraries were prepared using the Clontech SMART-Seq v4 Ultra Low Input RNA kit (Takara Bio) in combination with the NexteraXT DNA Library Preparation kit to append dual-indexed adapter sequences (Illumina). Libraries were validated by capillary electrophoresis on an Agilent 4200 TapeStation, pooled at equimolar concentrations, and sequenced on an Illumina NovaSeq6000 at 100SR, yielding 25 to 30 million reads per sample.

Raw reads were examined for quality issues using FastQC (https://www.bioinformatics.babraham.ac.uk/projects/fastqc/) to ensure library generation and sequencing are suitable for further analysis. Reads were aligned using STAR v2.7.3. Reads were aligned to Macaca mulatta genome. We used the macaque genome reference Macaca mulatta - MacaM (https://www.unmc.edu/rhesusgenechip/index.htm). DESeq2 was used to generate the normalized read count table based on their estimateSizeFactors() function with default parameters by calculating a pseudo-reference sample of the geometric means for each gene across all samples and then using the "median ratio" of each sample to the pseudo-reference as the sizeFactor for that sample. Differential expression at the gene level was performed by DESeq2 implemented in the DESeq2 R package, using counts per gene generated by a custom script that pulls out the library prep abundance estimation column into files, and read those files into DESeq2 with the DESeqDataSetFromHTSeqCount() function. A corrected p-value cut-off of 0.05 was used to assess genes that were significantly up-regulated or down-regulated at days 1, 7, 14, and 28 compared to baseline using the Benjamini-Hochberg (BH) method. Raw fastq files were uploaded to NCBI Gene Expression Omnibus (GEO) database under identifier GSE193264.

Pathway enrichment analysis

Gene set enrichment analysis (GSEA) and a compendium of databases of biological and immunological pathways were

used to test the longitudinal enrichment of pathways on days 1, 7, 14, and 28 post-vaccination compared to baseline on day 0. Genes were pre-ranked by fold change from the highest to the lowest, and GSEA was used to assess the enrichment of selected gene sets. Cytokines signaling, immune cell signatures, and molecular pathways were compiled from the M SigDB Hallmark, C2, C7, and C3 gene sets (https://www.gsea-msigdb.org/gsea/msigdb/collections.jsp), and the blood transcriptional modules (BTMs) (*36*). The GSEA Java desktop program was downloaded from the Broad Institute (https://www.broadinstitute.org/gsea/index.jsp) and used with GSEA Pre-Ranked module parameters (number of permutations: 1,000; enrichment statistic: weighted; 10≤ gene set size ≤5,000). Sample-level enrichment analysis (*37*) was used to investigate the enrichment of pathways in each animal. Briefly, the expression of all the genes in a specific pathway was averaged across samples and compared to the average expression of 1,000 randomly generated gene sets of the same size. The resulting Z score was then used to reflect the overall perturbation of each pathway in each individual sample. Pathways' sample level enrichment (SLE) scores were correlated with binding and neutralizing titers and B cell responses elicited by Ad26.COV2.S at the indicated days following vaccination, using Spearman correlation method.

Pharmacokinetics

To estimate antibody decay rate, the median values for $1x10^{11}$ and $5x10^{10}$ dose groups were plotted as a function of time post-vaccination, and the data were fitted to a biphasic decaying exponential model using the Curve Fitting Tool in MATLAB (version R_2021a). The half-life of the fast and slow phases of each decay model are indicated on the graphs (**fig. S1**). The day on which the decay function transitions from fast to slow decay is indicated in the blue dashed line for the $1x10^{11}$ dose and in the orange dashed line for the $5x10^{10}$ dose. The biphasic exponential decay is mathematically represented by:

$$V(t) = Ae^{\partial t} + Be^{\omega t} \quad (1)$$

where $V(t)$ is the antibody titer as a function of time; $A + B = V_0$, where V_0 the initial antibody titer at time t_0 (t = 0); and ∂, $\omega < 0$, are the decay rates of antibody titers.

To model each curve, time t_0 was taken to be the day post-vaccination when peak antibody responses were reached, and the remaining time points were adjusted accordingly. For example, for the $1x10^{11}$ vp dose ELISA titers, peak antibody responses were observed on day 28 post-vaccination. To model the antibody titers decay, day 28 was treated as t = 0 (t_0). This is due to the nature of the decaying exponential function: since t cannot take on negative values for our model, $t = 0$ is when the exponential decay reaches its maximum value. Therefore, the day that corresponds to peak antibody titers was treated as t_0. The data were then plotted as a function of days post-vaccination.

As per Eq. (1), the exponential function has two terms. The first term corresponds to the "fast" term, as this is when we observe a sharp decrease in antibody titers. The second term corresponds to the "slow" term, where the rate of antibody decay is lower. However, there is a given time denoted by a blue and orange dashed line in **fig. S1** where we observe a transition from fast decay to low decay, such that:

$$V(t^*) = Ae^{\partial t^*} = Be^{\omega t^*} \quad (2)$$

Furthermore, we characterized the antibody half-life for the fast and slow phases, defined as the time it takes for antibody titers to decrease by half of their initial value. To find the half-life, we equate Eq. (1) to $\frac{V_0}{2}$. For the fast phase, this is mathematically represented as:

$$\frac{V_0}{2} = Ae^{\partial t} + Be^{\omega t} \quad (3)$$

As such, solving Eq. 3 for t yields the half-life for the fast phase, which is indicated in the blue in **fig. S1** as $t_{1/2}$. Similarly, to determine the half-life for the slow phase, we can use:

$$\frac{V_0^*}{2} = Ae^{\partial t} + Be^{\omega t} \quad (4)$$

In Eq. 4, V_0^* is the amount of antibody titer at time t^* (given by solving Eq. 2) such that $V_0^* = V(t^*)$. Hence, solving for t in Eq. 4 gives the slow-phase half-life, which is indicated in orange in **fig. S1** as $t_{1/2}$.

Statistical analysis

All raw, individual-level data are presented in data files S1 to S6. Comparisons of immunologic data were performed using GraphPad Prism 8.4.2 (GraphPad Software). Comparison of data between groups was performed using two-sided Mann-Whitney tests or Wilcoxon signed-rank tests (for matched pairs). P values of less than 0.05 were considered significant. For differential expression gene (DEG) analyses, P values were corrected for multiple testing using the Benjamini-Hochberg (BH) method and a cut-off of 0.05. Pathway enrichment analysis was performed using R and was assessed using a false discovery rate (FDR) cut-off of 5%. Correlation analyses were performed using the cor.test R package, and statistical significance was assessed using two-sided Spearman rank-correlation tests.

SUPPLEMENTARY MATERIALS

www.science.org/doi/10.1126/scitranslmed.abm4996
Figs. S1 to S8
Data files S1 to S6
MDAR Reproducibility Checklist

REFERENCES AND NOTES

1. P. Abbink, A. A. Lemckert, B. A. Ewald, D. M. Lynch, M. Denholtz, S. Smits, L. Holterman, I. Damen, R. Vogels, A. R. Thorner, K. L. O'Brien, A. Carville, K. G. Mansfield, J. Goudsmit, M. J. Havenga, D. H. Barouch, Comparative seroprevalence and immunogenicity of six rare serotype recombinant adenovirus

vaccine vectors from subgroups B and D. *J. Virol.* **81**, 4654–4663 (2007). doi:10.1128/JVI.02696-06 Medline
2. R. Bos, L. Rutten, J. E. M. van der Lubbe, M. J. G. Bakkers, G. Hardenberg, F. Wegmann, D. Zuijdgeest, A. H. de Wilde, A. Koornneef, A. Verwilligen, D. van Manen, T. Kwaks, R. Vogels, T. J. Dalebout, S. K. Myeni, M. Kikkert, E. J. Snijder, Z. Li, D. H. Barouch, J. Vellinga, J. P. M. Langedijk, R. C. Zahn, J. Custers, H. Schuitemaker, Ad26 vector-based COVID-19 vaccine encoding a prefusion-stabilized SARS-CoV-2 Spike immunogen induces potent humoral and cellular immune responses. *NPJ Vaccines* **5**, 91 (2020). doi:10.1038/s41541-020-00243-x Medline
3. L. H. Tostanoski, F. Wegmann, A. J. Martinot, C. Loos, K. McMahan, N. B. Mercado, J. Yu, C. N. Chan, S. Bondoc, C. E. Starke, M. Nekorchuk, K. Busman-Sahay, C. Piedra-Mora, L. M. Wrijil, S. Ducat, J. Custers, C. Atyeo, S. Fischinger, J. S. Burke, J. Feldman, B. M. Hauser, T. M. Caradonna, E. A. Bondzie, G. Dagotto, M. S. Gebre, C. Jacob-Dolan, Z. Lin, S. H. Mahrokhian, F. Nampanya, R. Nityanandam, L. Pessaint, M. Porto, V. Ali, D. Benetiene, K. Tevi, H. Andersen, M. G. Lewis, A. G. Schmidt, D. A. Lauffenburger, G. Alter, J. D. Estes, H. Schuitemaker, R. Zahn, D. H. Barouch, Ad26 vaccine protects against SARS-CoV-2 severe clinical disease in hamsters. *Nat. Med.* **26**, 1694–1700 (2020). doi:10.1038/s41591-020-1070-6 Medline
4. N. B. Mercado, R. Zahn, F. Wegmann, C. Loos, A. Chandrashekar, J. Yu, J. Liu, L. Peter, K. McMahan, L. H. Tostanoski, X. He, D. R. Martinez, L. Rutten, R. Bos, D. van Manen, J. Vellinga, J. Custers, J. P. Langedijk, T. Kwaks, M. J. G. Bakkers, D. Zuijdgeest, S. K. Rosendahl Huber, C. Atyeo, S. Fischinger, J. S. Burke, J. Feldman, B. M. Hauser, T. M. Caradonna, E. A. Bondzie, G. Dagotto, M. S. Gebre, E. Hoffman, C. Jacob-Dolan, M. Kirilova, Z. Li, Z. Lin, S. H. Mahrokhian, L. F. Maxfield, F. Nampanya, R. Nityanandam, J. P. Nkolola, S. Patel, J. D. Ventura, K. Verrington, H. Wan, L. Pessaint, A. Van Ry, K. Blade, A. Strasbaugh, M. Cabus, R. Brown, A. Cook, S. Zouantchangadou, E. Teow, H. Andersen, M. G. Lewis, Y. Cai, B. Chen, A. G. Schmidt, R. K. Reeves, R. S. Baric, D. A. Lauffenburger, G. Alter, P. Stoffels, M. Mammen, J. Van Hoof, H. Schuitemaker, D. H. Barouch, Single-shot Ad26 vaccine protects against SARS-CoV-2 in rhesus macaques. *Nature* **586**, 583–588 (2020). doi:10.1038/s41586-020-2607-z Medline
5. L. Solforosi, H. Kuipers, M. Jongeneelen, S. K. Rosendahl Huber, J. E. M. van der Lubbe, L. Dekking, D. N. Czapska-Casey, A. Izquierdo Gil, M. R. M. Baert, J. Drijver, J. Vaneman, E. van Huizen, Y. Choi, J. Vreugdenhil, S. Kroos, A. H. de Wilde, E. Kourkouta, J. Custers, R. van der Vlugt, D. Veldman, J. Huizingh, K. Kaszas, T. J. Dalebout, S. K. Myeni, M. Kikkert, E. J. Snijder, D. H. Barouch, K. P. Böszörményi, M. A. Stammes, I. Kondova, E. J. Verschoor, B. E. Verstrepen, G. Koopman, P. Mooij, W. M. J. M. Bogers, M. van Heerden, L. Muchene, J. T. B. M. Tolboom, R. Roozendaal, B. Brandenburg, H. Schuitemaker, F. Wegmann, R. C. Zahn, Immunogenicity and efficacy of one and two doses of Ad26.COV2.S COVID vaccine in adult and aged NHP. *J. Exp. Med.* **218**, e20202756 (2021). doi:10.1084/jem.20202756 Medline
6. X. He, A. Chandrashekar, R. Zahn, F. Wegmann, J. Yu, N. B. Mercado, K. McMahan, A. J. Martinot, C. Piedra-Mora, S. Beecy, S. Ducat, R. Chamanza, S. R. Huber, M. van Heerden, L. van der Fits, E. N. Borducchi, M. Lifton, J. Liu, F. Nampanya, S. Patel, L. Peter, L. H. Tostanoski, L. Pessaint, A. Van Ry, B. Finneyfrock, J. Velasco, E. Teow, R. Brown, A. Cook, H. Andersen, M. G. Lewis, H. Schuitemaker, D. H. Barouch, Low-dose Ad26.COV2.S protection against SARS-CoV-2 challenge in rhesus macaques. *Cell* **184**, 3467–3473.e11 (2021). doi:10.1016/j.cell.2021.05.040 Medline
7. J. Sadoff, M. Le Gars, G. Shukarev, D. Heerwegh, C. Truyers, A. M. de Groot, J. Stoop, S. Tete, W. Van Damme, I. Leroux-Roels, P. J. Berghmans, M. Kimmel, P. Van Damme, J. de Hoon, W. Smith, K. E. Stephenson, S. C. De Rosa, K. W. Cohen, M. J. McElrath, E. Cormier, G. Scheper, D. H. Barouch, J. Hendriks, F. Struyf, M. Douoguih, J. Van Hoof, H. Schuitemaker, Interim Results of a Phase 1-2a Trial of Ad26.COV2.S Covid-19 Vaccine. *N. Engl. J. Med.* **384**, 1824–1835 (2021). doi:10.1056/NEJMoa2034201 Medline
8. K. E. Stephenson, M. Le Gars, J. Sadoff, A. M. de Groot, D. Heerwegh, C. Truyers, C. Atyeo, C. Loos, A. Chandrashekar, K. McMahan, L. H. Tostanoski, J. Yu, M. S. Gebre, C. Jacob-Dolan, Z. Li, S. Patel, L. Peter, J. Liu, E. N. Borducchi, J. P. Nkolola, M. Souza, C. S. Tan, R. Zash, B. Julg, R. R. Nathavitharana, R. L. Shapiro, A. A. Azim, C. D. Alonso, K. Jaegle, J. L. Ansel, D. G. Kanjilal, C. J. Guiney, C. Bradshaw, A. Tyler, T. Makoni, K. E. Yanosick, M. S. Seaman, D. A. Lauffenburger, G. Alter, F. Struyf, M. Douoguih, J. Van Hoof, H. Schuitemaker, D. H. Barouch, Immunogenicity of the Ad26.COV2.S Vaccine for COVID-19. *JAMA* **325**, 1535–1544 (2021). doi:10.1001/jama.2021.3645 Medline
9. J. Sadoff, G. Gray, A. Vandebosch, V. Cárdenas, G. Shukarev, B. Grinsztejn, P. A. Goepfert, C. Truyers, H. Fennema, B. Spiessens, K. Offergeld, G. Scheper, K. L. Taylor, M. L. Robb, J. Treanor, D. H. Barouch, J. Stoddard, M. F. Ryser, M. A. Marovich, K. M. Neuzil, L. Corey, N. Cauwenberghs, T. Tanner, K. Hardt, J. Ruiz-Guiñazú, M. Le Gars, H. Schuitemaker, J. Van Hoof, F. Struyf, M. Douoguih; ENSEMBLE Study Group, Safety and Efficacy of Single-Dose Ad26.COV2.S Vaccine against Covid-19. *N. Engl. J. Med.* **384**, 2187–2201 (2021). doi:10.1056/NEJMoa2101544 Medline
10. G. Alter, J. Yu, J. Liu, A. Chandrashekar, E. N. Borducchi, L. H. Tostanoski, K. McMahan, C. Jacob-Dolan, D. R. Martinez, A. Chang, T. Anioke, M. Lifton, J. Nkolola, K. E. Stephenson, C. Atyeo, S. Shin, P. Fields, I. Kaplan, H. Robins, F. Amanat, F. Krammer, R. S. Baric, M. Le Gars, J. Sadoff, A. M. de Groot, D. Heerwegh, F. Struyf, M. Douoguih, J. van Hoof, H. Schuitemaker, D. H. Barouch, Immunogenicity of Ad26.COV2.S vaccine against SARS-CoV-2 variants in humans. *Nature* **596**, 268–272 (2021). doi:10.1038/s41586-021-03681-2 Medline
11. K. E. Stephenson, M. Le Gars, J. Sadoff, A. M. de Groot, D. Heerwegh, C. Truyers, C. Atyeo, C. Loos, A. Chandrashekar, K. McMahan, L. H. Tostanoski, J. Yu, M. S. Gebre, C. Jacob-Dolan, Z. Li, S. Patel, L. Peter, J. Liu, E. N. Borducchi, J. P. Nkolola, M. Souza, C. S. Tan, R. Zash, B. Julg, R. R. Nathavitharana, R. L. Shapiro, A. A. Azim, C. D. Alonso, K. Jaegle, J. L. Ansel, D. G. Kanjilal, C. J. Guiney, C. Bradshaw, A. Tyler, T. Makoni, K. E. Yanosick, M. S. Seaman, D. A. Lauffenburger, G. Alter, F. Struyf, M. Douoguih, J. Van Hoof, H. Schuitemaker, D. H. Barouch, Immunogenicity of the Ad26.COV2.S Vaccine for COVID-19. *JAMA* **325**, 1535–1544 (2021). doi:10.1001/jama.2021.3645 Medline
12. D. H. Barouch, K. E. Stephenson, J. Sadoff, J. Yu, A. Chang, M. Gebre, K. McMahan, J. Liu, A. Chandrashekar, S. Patel, M. Le Gars, A. M. de Groot, D. Heerwegh, F. Struyf, M. Douoguih, J. van Hoof, H. Schuitemaker, Durable Humoral and Cellular Immune Responses 8 Months after Ad26.COV2.S Vaccination. *N. Engl. J. Med.* **385**, 951–953 (2021). doi:10.1056/NEJMc2108829 Medline
13. E. G. Levin, Y. Lustig, C. Cohen, R. Fluss, V. Indenbaum, S. Amit, R. Doolman, K. Asraf, E. Mendelson, A. Ziv, C. Rubin, L. Freedman, Y. Kreiss, G. Regev-Yochay, Waning Immune Humoral Response to BNT162b2 Covid-19 Vaccine over 6 Months. *N. Engl. J. Med.* **385**, e84 (2021). doi:10.1056/NEJMoa2114583 Medline
14. S. Cele, L. Jackson, D. S. Khoury, K. Khan, T. Moyo-Gwete, H. Tegally, J. E. San, D. Cromer, C. Scheepers, D. G. Amoako, F. Karim, M. Bernstein, G. Lustig, D. Archary, M. Smith, Y. Ganga, Z. Jule, K. Reedoy, S. H. Hwa, J. Giandhari, J. M. Blackburn, B. I. Gosnell, S. S. Abdool Karim, W. Hanekom, A. von Gottberg, J. N. Bhiman, R. J. Lessells, M. S. Moosa, M. P. Davenport, T. de Oliveira, P. L. Moore, A. Sigal; NGS-SA; COMMIT-KZN Team, Omicron extensively but incompletely escapes Pfizer BNT162b2 neutralization. *Nature* (2021). doi:10.1038/s41586-021-04387-1 Medline
15. L. Liu, S. Iketani, Y. Guo, J. F. Chan, M. Wang, L. Liu, Y. Luo, H. Chu, Y. Huang, M. S. Nair, J. Yu, K. K. Chik, T. T. Yuen, C. Yoon, K. K. To, H. Chen, M. T. Yin, M. E. Sobieszczyk, Y. Huang, H. H. Wang, Z. Sheng, K. Y. Yuen, D. D. Ho, Striking Antibody Evasion Manifested by the Omicron Variant of SARS-CoV-2. *Nature* (2021). doi:10.1038/s41586-021-04388-0 Medline
16. I. Nemet, L. Kliker, Y. Lustig, N. Zuckerman, O. Erster, C. Cohen, Y. Kreiss, S. Alroy-Preis, G. Regev-Yochay, E. Mendelson, M. Mandelboim, Third BNT162b2 Vaccination Neutralization of SARS-CoV-2 Omicron Infection. *N. Engl. J. Med.* **386**, 492–494 (2022). Medline
17. P. Mlcochova, S. A. Kemp, M. S. Dhar, G. Papa, B. Meng, I. A. T. M. Ferreira, R. Datir, D. A. Collier, A. Albecka, S. Singh, R. Pandey, J. Brown, J. Zhou, N. Goonawardane, S. Mishra, C. Whittaker, T. Mellan, R. Marwal, M. Datta, S. Sengupta, K. Ponnusamy, V. S. Radhakrishnan, A. Abdullahi, O. Charles, P. Chattopadhyay, P. Devi, D. Caputo, T. Peacock, C. Wattal, N. Goel, A. Satwik, R. Vaishya, M. Agarwal, A. Mavousian, J. H. Lee, J. Bassi, C. Silacci-Fegni, C. Saliba, D. Pinto, T. Irie, I. Yoshida, W. L. Hamilton, K. Sato, S. Bhatt, S. Flaxman, L. C. James, D. Corti, L. Piccoli, W. S. Barclay, P. Rakshit, A. Agrawal, R. K. Gupta; Indian SARS-CoV-2 Genomics Consortium (INSACOG); Genotype to Phenotype Japan (G2P-Japan) Consortium; CITIID-NIHR BioResource COVID-19 Collaboration, SARS-CoV-2 B.1.617.2 Delta variant replication and immune evasion. *Nature* **599**, 114–119 (2021). doi:10.1038/s41586-021-03944-y Medline

18. T. Aydillo, A. Rombauts, D. Stadlbauer, S. Aslam, G. Abelenda-Alonso, A. Escalera, F. Amanat, K. Jiang, F. Krammer, J. Carratala, A. García-Sastre, Immunological imprinting of the antibody response in COVID-19 patients. *Nat. Commun.* **12**, 3781 (2021). doi:10.1038/s41467-021-23977-1 Medline
19. A. Choi, M. Koch, K. Wu, L. Chu, L. Ma, A. Hill, N. Nunna, W. Huang, J. Oestreicher, T. Colpitts, H. Bennett, H. Legault, Y. Paila, B. Nestorova, B. Ding, D. Montefiori, R. Pajon, J. M. Miller, B. Leav, A. Carfi, R. McPhee, D. K. Edwards, Safety and immunogenicity of SARS-CoV-2 variant mRNA vaccine boosters in healthy adults: An interim analysis. *Nat. Med.* **27**, 2025–2031 (2021). doi:10.1038/s41591-021-01527-y Medline
20. A. Chandrashekar, J. Liu, A. J. Martinot, K. McMahan, N. B. Mercado, L. Peter, L. H. Tostanoski, J. Yu, Z. Maliga, M. Nekorchuk, K. Busman-Sahay, M. Terry, L. M. Wrijil, S. Ducat, D. R. Martinez, C. Atyeo, S. Fischinger, J. S. Burke, M. D. Slein, L. Pessaint, A. Van Ry, J. Greenhouse, T. Taylor, K. Blade, A. Cook, B. Finneyfrock, R. Brown, E. Teow, J. Velasco, R. Zahn, F. Wegmann, P. Abbink, E. A. Bondzie, G. Dagotto, M. S. Gebre, X. He, C. Jacob-Dolan, N. Kordana, Z. Li, M. A. Lifton, S. H. Mahrokhian, L. F. Maxfield, R. Nityanandam, J. P. Nkolola, A. G. Schmidt, A. D. Miller, R. S. Baric, G. Alter, P. K. Sorger, J. D. Estes, H. Andersen, M. G. Lewis, D. H. Barouch, SARS-CoV-2 infection protects against rechallenge in rhesus macaques. *Science* **369**, 812–817 (2020). doi:10.1126/science.abc4776 Medline
21. J. Yu, L. H. Tostanoski, N. B. Mercado, K. McMahan, J. Liu, C. Jacob-Dolan, A. Chandrashekar, C. Atyeo, D. R. Martinez, T. Anioke, E. A. Bondzie, A. Chang, S. Gardner, V. M. Giffin, D. L. Hope, F. Nampanya, J. Nkolola, S. Patel, O. Sanborn, D. Sellers, H. Wan, T. Hayes, K. Bauer, L. Pessaint, D. Valentin, Z. Flinchbaugh, R. Brown, A. Cook, D. Bueno-Wilkerson, E. Teow, H. Andersen, M. G. Lewis, A. J. Martinot, R. S. Baric, G. Alter, F. Wegmann, R. Zahn, H. Schuitemaker, D. H. Barouch, Protective efficacy of Ad26.COV2.S against SARS-CoV-2 B.1.351 in macaques. *Nature* **596**, 423–427 (2021). doi:10.1038/s41586-021-03732-8 Medline
22. J. Yu, L. H. Tostanoski, L. Peter, N. B. Mercado, K. McMahan, S. H. Mahrokhian, J. P. Nkolola, J. Liu, Z. Li, A. Chandrashekar, D. R. Martinez, C. Loos, C. Atyeo, S. Fischinger, J. S. Burke, M. D. Slein, Y. Chen, A. Zuiani, F. J. N. Lelis, M. Travers, S. Habibi, L. Pessaint, A. Van Ry, K. Blade, R. Brown, A. Cook, B. Finneyfrock, A. Dodson, E. Teow, J. Velasco, R. Zahn, F. Wegmann, E. A. Bondzie, G. Dagotto, M. S. Gebre, X. He, C. Jacob-Dolan, M. Kirilova, N. Kordana, Z. Lin, L. F. Maxfield, F. Nampanya, R. Nityanandam, J. D. Ventura, H. Wan, Y. Cai, B. Chen, A. G. Schmidt, D. R. Wesemann, R. S. Baric, G. Alter, H. Andersen, M. G. Lewis, D. H. Barouch, DNA vaccine protection against SARS-CoV-2 in rhesus macaques. *Science* **369**, 806–811 (2020). doi:10.1126/science.abc6284 Medline
23. J. S. Turner, W. Kim, E. Kalaidina, C. W. Goss, A. M. Rauseo, A. J. Schmitz, L. Hansen, A. Haile, M. K. Klebert, I. Pusic, J. A. O'Halloran, R. M. Presti, A. H. Ellebedy, SARS-CoV-2 infection induces long-lived bone marrow plasma cells in humans. *Nature* **595**, 421–425 (2021). doi:10.1038/s41586-021-03647-4 Medline
24. R. A. Manz, A. Thiel, A. Radbruch, Lifetime of plasma cells in the bone marrow. *Nature* **388**, 133–134 (1997). doi:10.1038/40540 Medline
25. E. Hammarlund, M. W. Lewis, S. G. Hansen, L. I. Strelow, J. A. Nelson, G. J. Sexton, J. M. Hanifin, M. K. Slifka, Duration of antiviral immunity after smallpox vaccination. *Nat. Med.* **9**, 1131–1137 (2003). doi:10.1038/nm917 Medline
26. P. Martinez-Murillo, L. Pramanik, C. Sundling, K. Hultenby, P. Wretenberg, M. Spångberg, G. B. Karlsson Hedestam, CD138 and CD31 Double-Positive Cells Comprise the Functional Antibody-Secreting Plasma Cell Compartment in Primate Bone Marrow. *Front. Immunol.* **7**, 242 (2016). doi:10.3389/fimmu.2016.00242 Medline
27. C. Jacob-Dolan, J. Feldman, K. McMahan, J. Yu, R. Zahn, F. Wegmann, H. Schuitemaker, A. G. Schmidt, D. H. Barouch, Coronavirus-Specific Antibody Cross Reactivity in Rhesus Macaques Following SARS-CoV-2 Vaccination and Infection. *J. Virol.* **95**, JVI.00117-21 (2021). doi:10.1128/JVI.00117-21 Medline
28. A. W. Chung, M. P. Kumar, K. B. Arnold, W. H. Yu, M. K. Schoen, L. J. Dunphy, T. J. Suscovich, N. Frahm, C. Linde, A. E. Mahan, M. Hoffner, H. Streeck, M. E. Ackerman, M. J. McElrath, H. Schuitemaker, M. G. Pau, L. R. Baden, J. H. Kim, N. L. Michael, D. H. Barouch, D. A. Lauffenburger, G. Alter, Dissecting Polyclonal Vaccine-Induced Humoral Immunity against HIV Using Systems Serology. *Cell* **163**, 988–998 (2015). doi:10.1016/j.cell.2015.10.027 Medline
29. A. Y. Collier, J. Yu, K. McMahan, J. Liu, A. Chandrashekar, J. S. Maron, C. Atyeo, D. R. Martinez, J. L. Ansel, R. Aguayo, M. Rowe, C. Jacob-Dolan, D. Sellers, J. Barrett, K. Ahmad, T. Anioke, H. VanWyk, S. Gardner, O. Powers, E. A. Bondzie, H. Wan, R. S. Baric, G. Alter, M. R. Hacker, D. H. Barouch, Differential Kinetics of Immune Responses Elicited by Covid-19 Vaccines. *N. Engl. J. Med.* **385**, 2010–2012 (2021). doi:10.1056/NEJMc2115596 Medline
30. K. McMahan, J. Yu, N. B. Mercado, C. Loos, L. H. Tostanoski, A. Chandrashekar, J. Liu, L. Peter, C. Atyeo, A. Zhu, E. A. Bondzie, G. Dagotto, M. S. Gebre, C. Jacob-Dolan, Z. Li, F. Nampanya, S. Patel, L. Pessaint, A. Van Ry, K. Blade, J. Yalley-Ogunro, M. Cabus, R. Brown, A. Cook, E. Teow, H. Andersen, M. G. Lewis, D. A. Lauffenburger, G. Alter, D. H. Barouch, Correlates of protection against SARS-CoV-2 in rhesus macaques. *Nature* **590**, 630–634 (2021). doi:10.1038/s41586-020-03041-6 Medline
31. J. Sadoff, M. Le Gars, V. Cardenas, G. Shukarev, N. Vaissiere, D. Heerwegh, C. Truyers, A. M. de Groot, G. Scheper, J. Hendriks, J. Ruiz-Guiñazú, F. Struyf, J. Van Hoof, M. Douoguih, H. Schuitemaker, Durability of antibody responses elicited by a single dose of Ad26.COV2.S and substantial increase following late boosting. *medRxiv*, 2021.2008.2025.21262569 (2021).
32. J. Sadoff, M. Le Gars, G. Shukarev, D. Heerwegh, C. Truyers, A. M. de Groot, J. Stoop, S. Tete, W. Van Damme, I. Leroux-Roels, P. J. Berghmans, M. Kimmel, P. Van Damme, J. de Hoon, W. Smith, K. E. Stephenson, S. C. De Rosa, K. W. Cohen, M. J. McElrath, E. Cormier, G. Scheper, D. H. Barouch, J. Hendriks, F. Struyf, M. Douoguih, J. Van Hoof, H. Schuitemaker, Interim Results of a Phase 1-2a Trial of Ad26.COV2.S Covid-19 Vaccine. *N. Engl. J. Med.* **384**, 1824–1835 (2021). doi:10.1056/NEJMoa2034201 Medline
33. L. R. Baden, S. R. Walsh, M. S. Seaman, R. P. Tucker, K. H. Krause, A. Patel, J. A. Johnson, J. Kleinjan, K. E. Yanosick, J. Perry, E. Zablowsky, P. Abbink, L. Peter, M. J. Iampietro, A. Cheung, M. G. Pau, M. Weijtens, J. Goudsmit, E. Swann, M. Wolff, H. Loblein, R. Dolin, D. H. Barouch, First-in-human evaluation of the safety and immunogenicity of a recombinant adenovirus serotype 26 HIV-1 Env vaccine (IPCAVD 001). *J. Infect. Dis.* **207**, 240–247 (2013). doi:10.1093/infdis/jis670 Medline
34. D. H. Barouch, J. Liu, L. Peter, P. Abbink, M. J. Iampietro, A. Cheung, G. Alter, A. Chung, A. S. Dugast, N. Frahm, M. J. McElrath, H. Wenschuh, U. Reimer, M. S. Seaman, M. G. Pau, M. Weijtens, J. Goudsmit, S. R. Walsh, R. Dolin, L. R. Baden, Characterization of humoral and cellular immune responses elicited by a recombinant adenovirus serotype 26 HIV-1 Env vaccine in healthy adults (IPCAVD 001). *J. Infect. Dis.* **207**, 248–256 (2013). doi:10.1093/infdis/jis671 Medline
35. M. Koutsakos, A. K. Wheatley, L. Loh, E. B. Clemens, S. Sant, S. Nüssing, A. Fox, A. W. Chung, K. L. Laurie, A. C. Hurt, S. Rockman, M. Lappas, T. Loudovaris, S. I. Mannering, G. P. Westall, M. Elliot, S. G. Tangye, L. M. Wakim, S. J. Kent, T. H. O. Nguyen, K. Kedzierska, Circulating T_{FH} cells, serological memory, and tissue compartmentalization shape human influenza-specific B cell immunity. *Sci. Transl. Med.* **10**, eaan8405 (2018). doi:10.1126/scitranslmed.aan8405 Medline
36. S. Li, N. Rouphael, S. Duraisingham, S. Romero-Steiner, S. Presnell, C. Davis, D. S. Schmidt, S. E. Johnson, A. Milton, G. Rajam, S. Kasturi, G. M. Carlone, C. Quinn, D. Chaussabel, A. K. Palucka, M. J. Mulligan, R. Ahmed, D. S. Stephens, H. I. Nakaya, B. Pulendran, Molecular signatures of antibody responses derived from a systems biology study of five human vaccines. *Nat. Immunol.* **15**, 195–204 (2014). doi:10.1038/ni.2789 Medline
37. G. Gundem, N. Lopez-Bigas, Sample-level enrichment analysis unravels shared stress phenotypes among multiple cancer types. *Genome Med.* **4**, 28 (2012). doi:10.1186/gm327 Medline

Acknowledgments: We thank Shivani Patel, Owen Sanborn, Haley VanWyk, Alessandro Colarusso, Aiquan Chang, Makda Gebre, Benjamin Chung, Olivia Powers, Mazuba Siamatu for generous advice, assistance, and reagents. We thank Mesoscale Discovery for the ECLA kits. **Funding:** We acknowledge support from Janssen Vaccines & Prevention BV, the National Institutes of Health (CA260476 to D.H.B.), the Ragon Institute of MGH, MIT, and Harvard, the Musk Foundation, the Massachusetts Consortium on Pathogen Readiness (MassCPR), and the Biomedical Advanced Research and Development Authority (BARDA; HHSO100201700018C to D.H.B.). **Author Contributions:** D.H.B., F.W., R.Z., and H.S. designed the study. X.H., M.A., A.C., J.Y., K.M., C.J.D., J.S.M., C.A., H.W., D.S., J.L., M.L., S.G., E.A.B., J.B., K.A., T.A., and G.A. performed the immunologic assays. F.W., R.Z., and H.S. provided the vaccines. J.Y.O., J.M., A.G., H.A., and M.G.L. led the clinical care of the animals. X.H., M.A., and D.H.B. wrote the paper with all co-authors. **Competing Interests:** D.H.B. is a co-inventor on provisional

vaccine patents (63/121,482; 63/133,969; 63/135,182 "Compositions and Methods for Preventing and Treating Coronavirus Infection - SARS-COV-2 Vaccines"). F.W., R.Z., and H.S. are employees of Janssen Pharmaceuticals and co-inventors on provisional vaccine patents (63/121,482; 63/133,969; 63/135,182 "Compositions and Methods for Preventing and Treating Coronavirus Infection - SARS-COV-2 Vaccines"). **Data Availability Statement:** All data are available in the manuscript and the supplementary material. Materials, including vaccines, will be made available by contacting D.H.B. (dbarouch@bidmc.harvard.edu) and completion of a material transfer agreement. Raw fastq files were uploaded to NCBI Gene Expression Omnibus (GEO) database under identifier GSE193264. This work is licensed under a Creative Commons Attribution 4.0 International (CC BY 4.0) license, which permits unrestricted use, distribution, and reproduction in any medium, provided the original work is properly cited. To view a copy of this license, visit https://creativecommons.org/licenses/by/4.0/. This license does not apply to figures/photos/artwork or other content included in the article that is credited to a third party; obtain authorization from the rights holder before using this material.

Submitted 21 December 2021
Accepted 11 February 2022
Published First Release 22 February 2022
10.1126/scitranslmed.abm4996

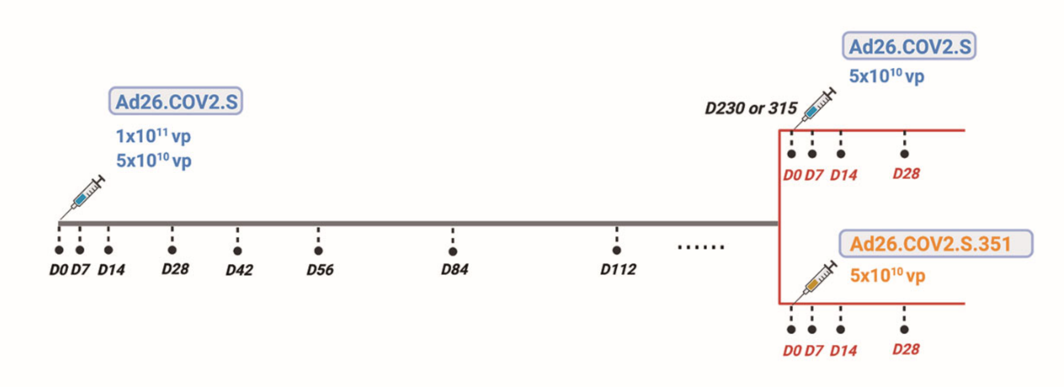

Fig. 1. Study schema. Twenty rhesus macaques were immunized with $1x10^{11}$ vp (n=10) or $5x10^{10}$ vp (n=10) Ad26.COV2.S. The study start was staggered, and half of the animals in each dose group were followed for 230 or 315 days. Blood was collected longitudinally from day 0 through day 230 or 315 post-immunization to evaluate the durability of single-dose Ad26.COV2.S. On day 230 or 315, all the macaques received a boost immunization with $5x10^{10}$ vp Ad26.COV2.S (n=10) or Ad26.COV2.S.351 (n=10). vp, viral particles.

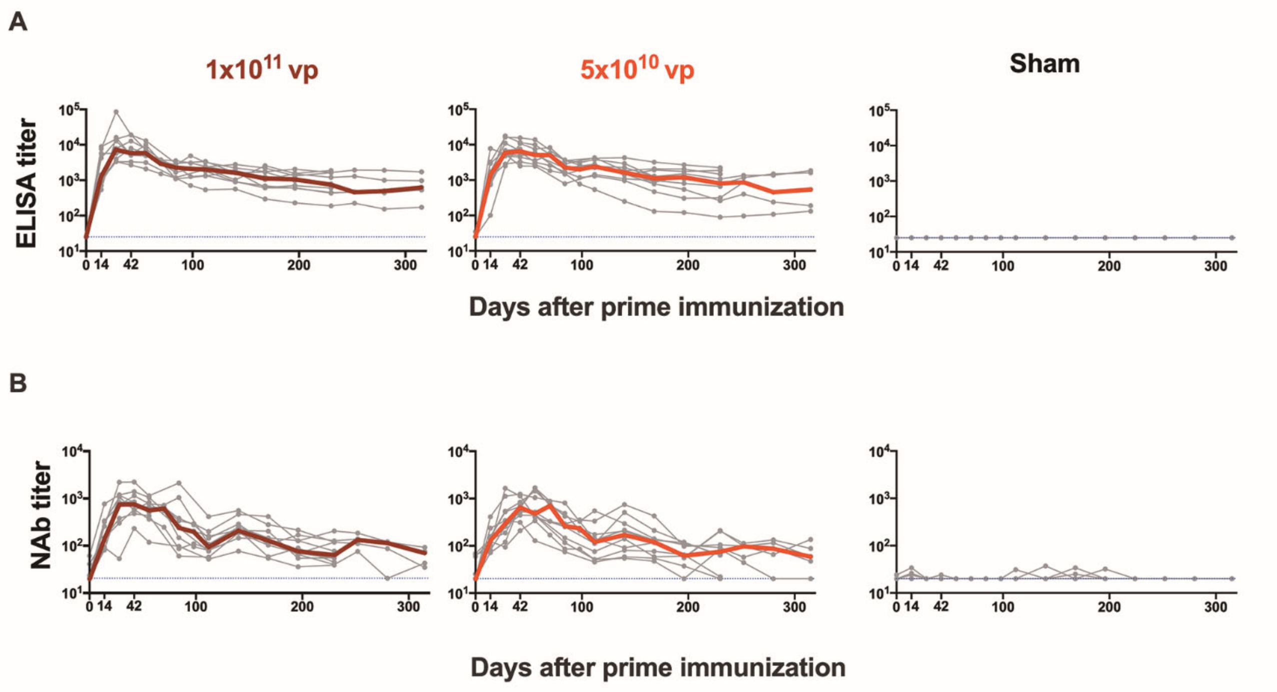

Fig. 2. SARS-CoV-2 specific antibody responses are durable following single-shot Ad26.COV2.S vaccination. (A) SARS-CoV-2 WA1/2020 RBD-specific binding antibody responses were measured by ELISA against SARS-CoV-2 in rhesus macaques following single-shot immunization with $1x10^{11}$ vp (n=10) or $5x10^{10}$ vp (n=10) Ad26.COV2.S or sham (n=4). (B) Pseudovirus neutralizing antibody (NAb) responses against SARS-CoV-2 WA1/2020 were measured. Bold lines reflect median values. Dotted lines reflect lower limits of quantitation.

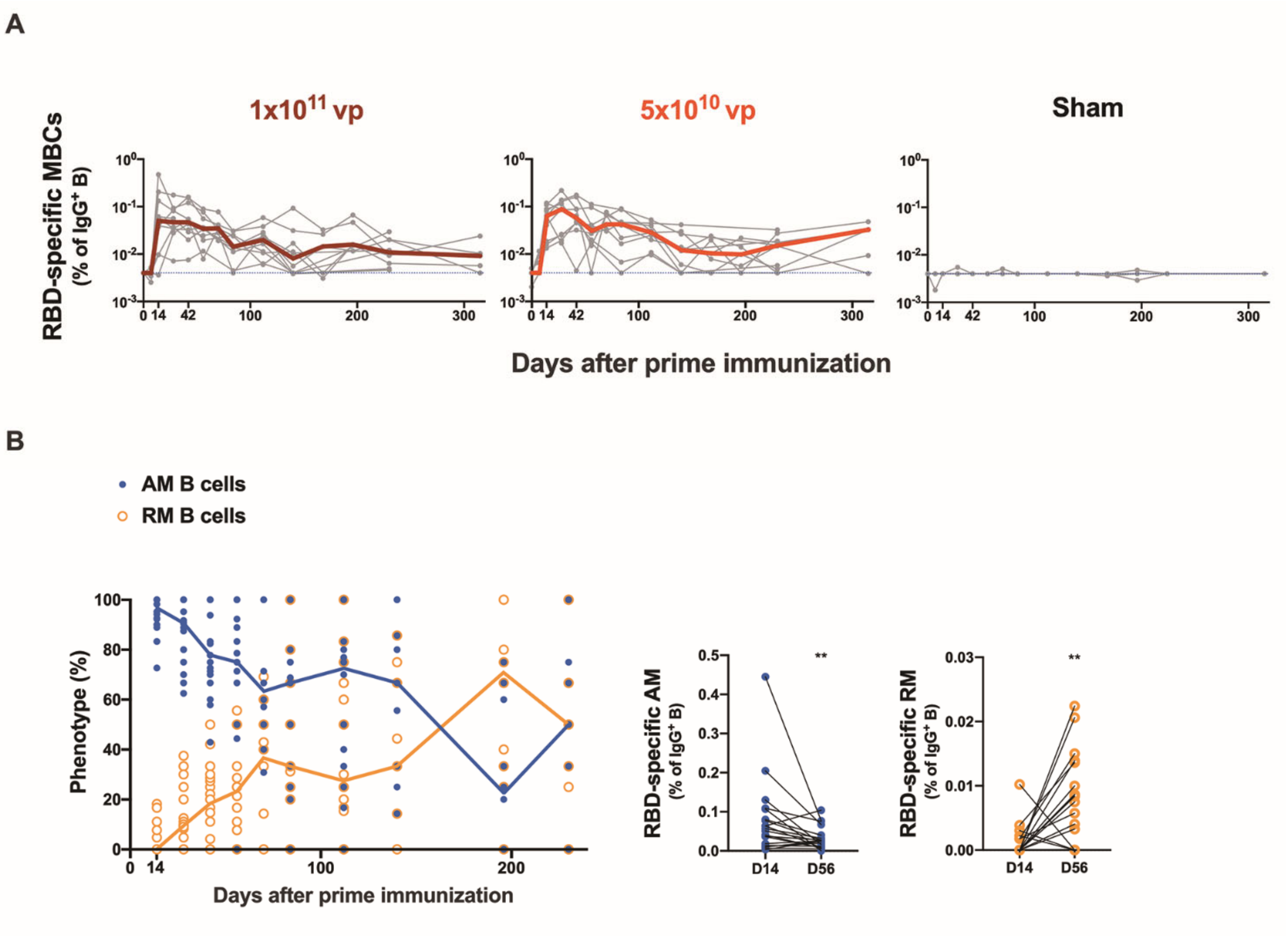

Fig. 3. RBD-specific memory B cell responses are sustained following single-shot Ad26.COV2.S vaccination. (**A**) Longitudinal analysis of WA1/2020 RBD-specific IgG+ memory B cells (MBCs) in macaques is shown following single-shot immunization with $1x10^{11}$ vp (n=10) or $5x10^{10}$ vp (n=10) Ad26.COV2.S or sham (n=4). Bold lines reflect median values. Dotted lines reflect lower limits of quantitation. (**B**) Longitudinal WA1/2020 RBD-specific AM (blue) and RM B cells (orange) proportions are shown following initial vaccination. Blue and orange lines reflect median values for AM and RM B cells, respectively. SARS-CoV-2 RBD-specific AM (blue) and RM (orange) B cells in IgG+ B cells are shown on days 14 and 56 following prime immunization. ** P<0.01, two-sided Wilcoxon signed-rank test.

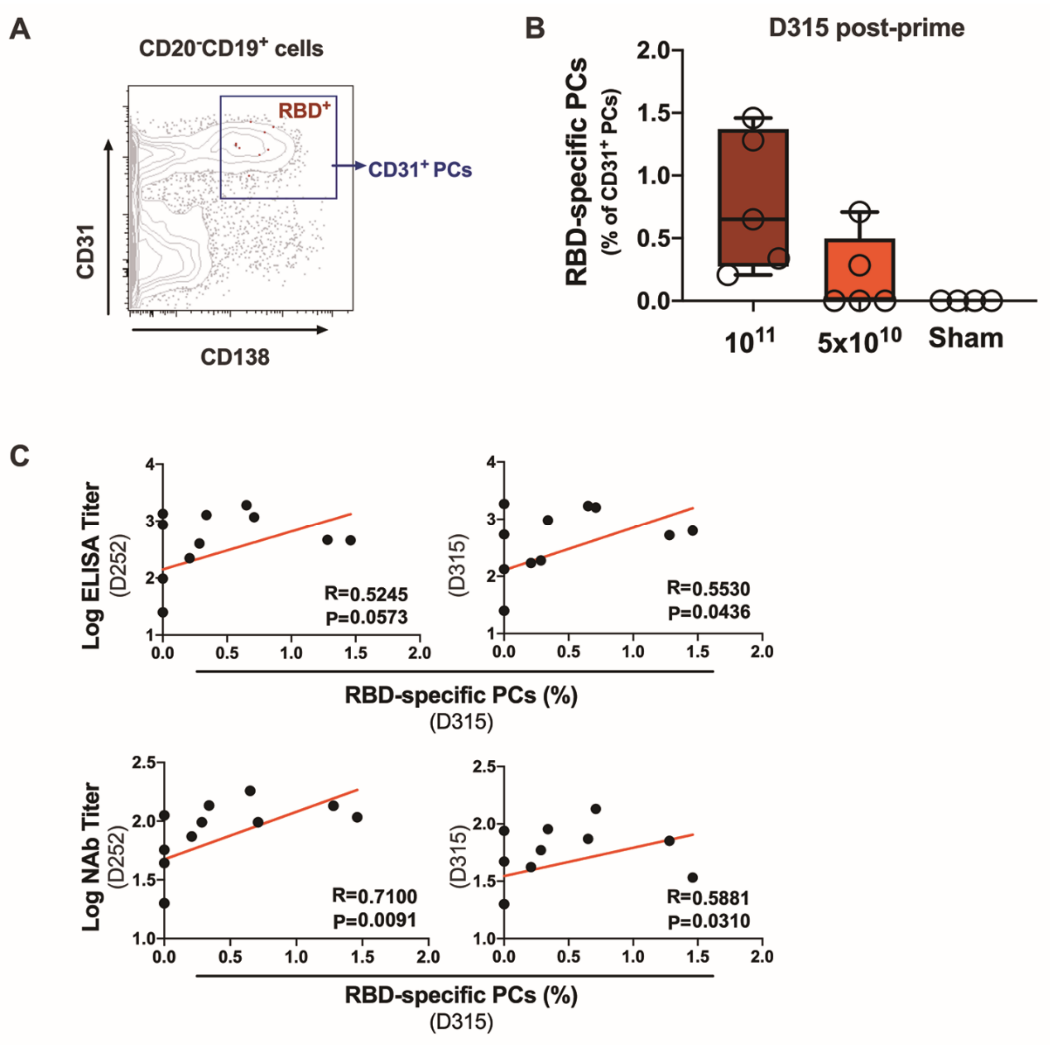

Fig. 4. RBD-specific plasma cells in bone marrow persist following single-shot Ad26.COV2.S vaccination. (A) Representative flow cytometry plot show WA1/2020 RBD-specific plasma cells (PCs) in the $CD138^+CD31^+$ population gated from $CD20^-CD19^+$ cells in bone marrow isolated from vaccinated macaques. (B) Frequencies of RBD-specific $CD31^+$ PCs in bone marrow in macaques on day 315 following single-shot immunization with $1x10^{11}$ vp (n=5) or $5x10^{10}$ vp (n=5) Ad26.COV2.S or sham (n=4). Box and whisker plots indicate interquartile ranges (boxes), medians (horizontal lines), and range (whiskers). (C) Correlation of RBD-specific PCs on day 315 with binding (upper panels) or neutralizing (lower panels) antibody responses on day 252 (left panels) or day 315 (right panels) following vaccination. Red lines reflect best linear fits. P and R values reflect two-sided Spearman rank-correlation tests.

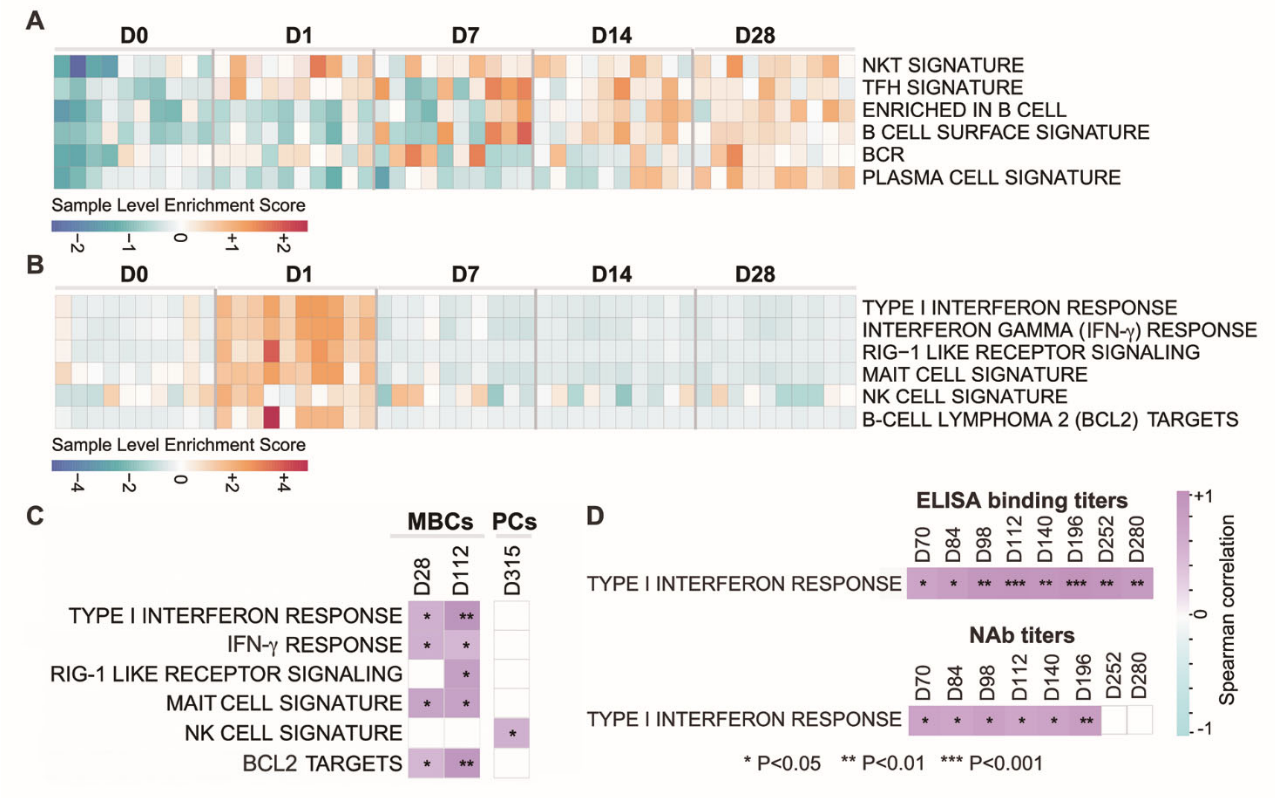

Fig. 5. Innate immune signatures correlated with durable antibody responses. (**A**) Heatmaps show the row scaled samples level enrichment (SLE) scores of natural killer T (NKT), T follicular helper (TFH), B cell, B cell receptor, and plasma cell signatures on days 0, 1, 7, 14, and 28 following single-shot Ad26.COV2.S vaccination. (**B**) Heatmaps show the row scaled SLE scores of innate pathways up-regulated on day 1 following single-shot Ad26.COV2.S vaccination. (**C**) Correlation between SLE scores of pathways up-regulated on day 1 following single-shot Ad26.COV2.S vaccination and RBD-specific memory B cells (MBCs) or plasma cells (PCs) following vaccination. Columns correspond to individual animals, and rows correspond to individual pathways. Purple color gradient indicates the Z score normalized SLE of each pathway across animals, measured as the mean expression of pathways leading genes (BH-adjusted p<0.05) for each individual animal. (**D**) Spearman correlation of type I interferon signature SLE score and binding or neutralizing antibody titers is shown for multiple time points. Spearman correlation coefficients range from cyan (negative correlation) to purple (positive correlation). Blank squares indicate no significance. * P<0.05, **P<0.01, ***P<0.001.

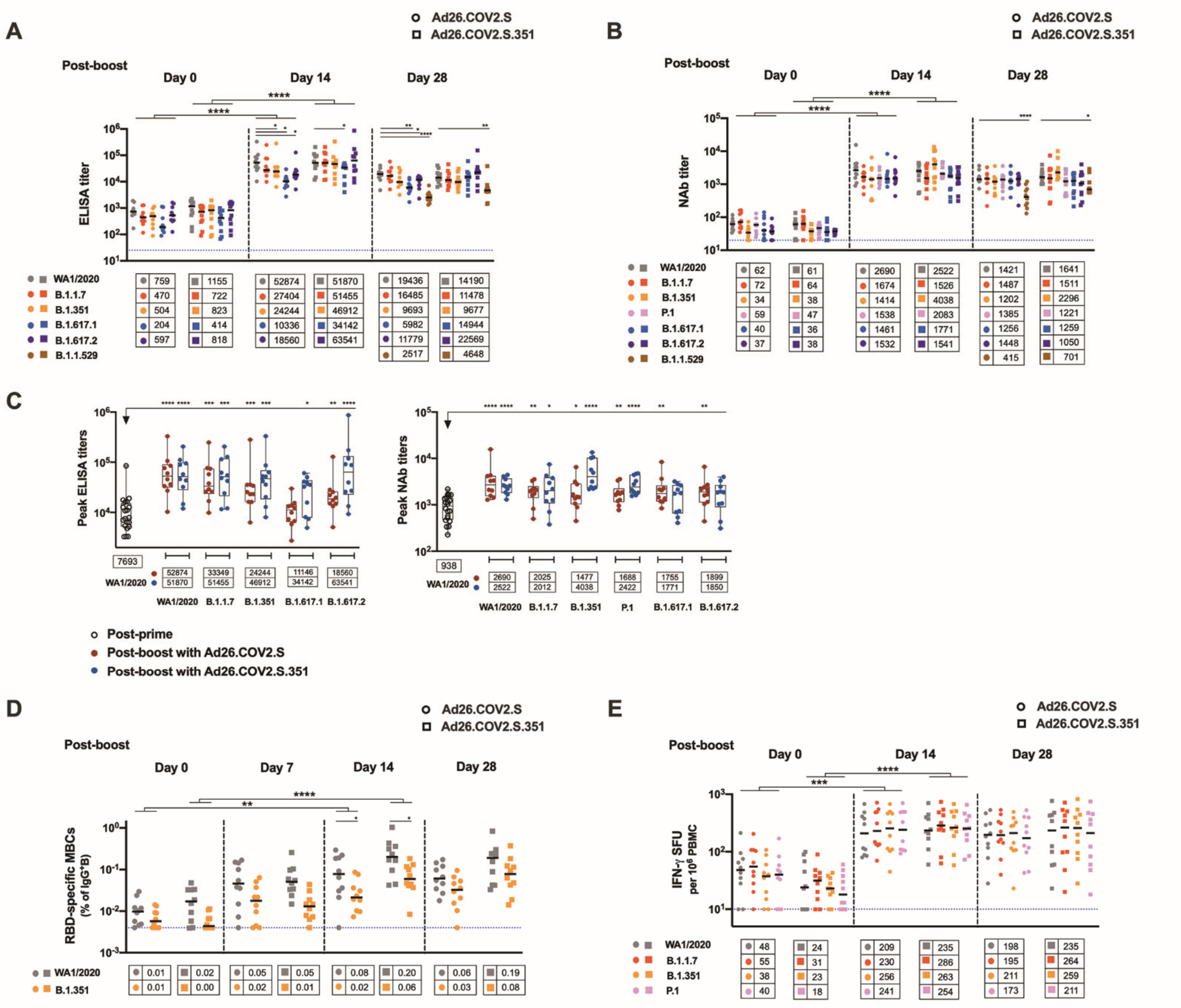

Fig. 6. SARS-CoV-2 specific antibody and T cell responses are enhanced following boost immunization. (A) RBD-specific binding antibody responses and (B) NAb responses against SARS-CoV-2 variants WA1/2020 (ancestral), B.1.1.7 (alpha), B.1.351 (beta), P.1 (gamma), B.1.617.1 (kappa), and B.1.617.2 (delta) are shown for samples collected at days 0, 14, and 28 following the boost immunization with $5x10^{10}$ vp of Ad26.COV2.S (n=10) or Ad26.COV2.S.351 (n=10). Responses to B.1.1.529 (Omicron) are also shown for samples collected on day 28. Antibody responses to WA1/2020 were compared with responses to variants at each time point. Antibody responses against variants from day 0 were compared with responses from day 14 following the boost immunization. (C) Comparisons of peak responses against WA1/2020 following the initial single-shot Ad26.COV2.S immunization and peak responses against WA1/2020, B.1.1.7, B.1.351, P.1, B.1.617.1, and B.1.617.2 following the boost immunization with $5x10^{10}$ vp of Ad26.COV2.S or Ad26.COV2.S.351 are shown. Box and whisker plots indicate interquartile ranges (boxes), medians (horizontal lines), and range (whiskers). (D) RBD-specific memory B cells (MBCs) and (E) T cell responses by pooled peptide IFN-γ ELISPOT assays were measured on samples collected at days 0, 14, and 28 following the boost immunization. B cell responses to WA1/2020 were compared with responses to the beta variant at each time point. B cell and T cell responses against variants from day 0 were compared with responses from day 14 following the boost immunization. Bold horizontal lines and numbers in boxes reflect median values. Dotted lines reflect lower limits of quantitation. * $P<0.05$, ** $P<0.01$, *** $P<0.001$, **** $P<0.0001$, two-sided Mann-Whitney tests for comparisons between WA1/2020 group and individual variants at each time point (smaller asterisks), as well as for comparisons between individual variants on days 0 and 14 (larger asterisks reflecting P values for the individual variants compared between two groups). SFU, spot-forming unit.
