# Supplementary material for: A homologous or variant booster vaccine after Ad26.COV2.S immunization enhances SARS-CoV-2–specific immune responses in rhesus macaques
Source: Sci Transl Med. 2022 Mar 30;14(638):eabm4996. doi: 10.1126/scitranslmed.abm4996 (PMC9802654; doi:10.1126/scitranslmed.abm4996)
Supplement: Supplementary file 2 — Figs. S1 to S8 [file scitranslmed.abm4996_sm.pdf]

Science Translational Medicine
AAAS

# Supplementary Materials for

## A homologous or variant booster vaccine after Ad26.COV2.S immunization enhances SARS-CoV-2–specific immune responses in rhesus macaques

Xuan He *et al.*

Corresponding author: Dan H. Barouch, dbarouch@bidmc.harvard.edu

*Sci. Transl. Med.* **14**, eabm4996 (2022)
DOI: 10.1126/scitranslmed.abm4996

**The PDF file includes:**

Figs. S1 to S8
Legends for data files S1 to S6

**Other Supplementary Material for this manuscript includes the following:**

Data files S1 to S6
MDAR Reproducibility Checklist

# Supplementary Figures

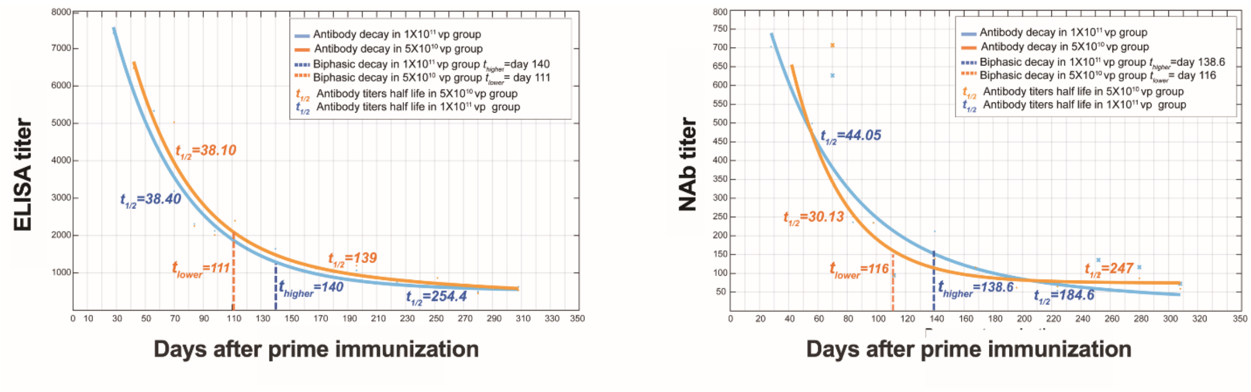


**Fig. S1. Biphasic decay of antibody responses following single-shot Ad26.COV2.S vaccination.**

Half-life calculation for WA1/2020 receptor-binding domain (RBD)-specific binding (left) and neutralizing (right) antibody responses in rhesus macaques following immunization with $1x10^{11}$ vp (n=10) or $5x10^{10}$ vp (n=10) Ad26.COV2.S. The median values for $1x10^{11}$ vp (blue) and $5x10^{10}$ vp (orange) groups were plotted as a function of time post-vaccination, and the data were fitted to a biphasic decaying exponential model using the curve fitting tool in MATLAB. The half-life of the fast and the slow phases of each decay model are indicated on the graphs by $t_{1/2}$, for the higher dose in blue and for the lower dose in orange. The day on which the decay function transitions from the fast to the slow phase is indicated as $t_{higher}$ in blue dashed line for the $1x10^{11}$ vp dose and as $t_{lower}$ in orange dashed line for the $5x10^{10}$ vp dose. ELISA, enzyme-linked immunosorbent assay; NAb, neutralizing antibody; vp, viral particles.

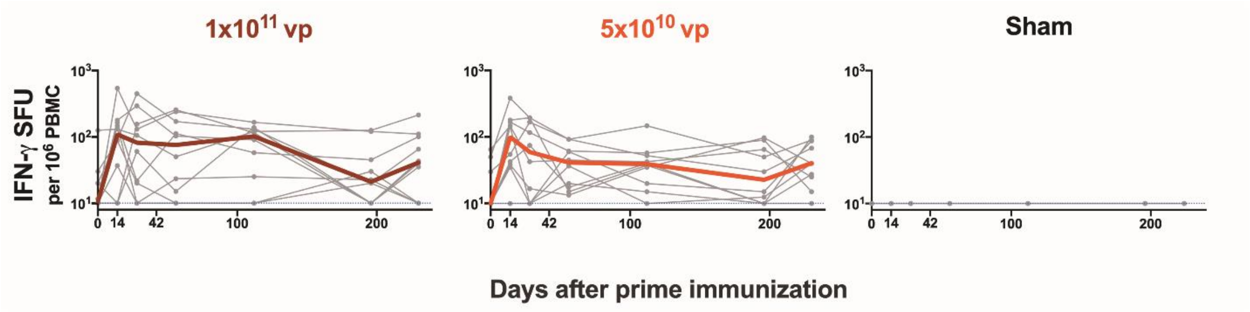


**Fig. S2. T cell responses following single-shot Ad26.COV2.S vaccination.** T cell responses to pooled spike (WA1/2020) peptides were assessed by IFN-γ ELISPOT assays following immunization with $1x10^{11}$ vp (n=10) or $5x10^{10}$ vp (n=10) Ad26.COV2.S or sham (n=4). Bold lines reflect median values. Dotted lines reflect lower limits of quantitation. IFN, interferon; ELISPOT; enzyme-linked immune absorbent spot; SFU, spot-forming units; PBMC, peripheral blood mononuclear cells.

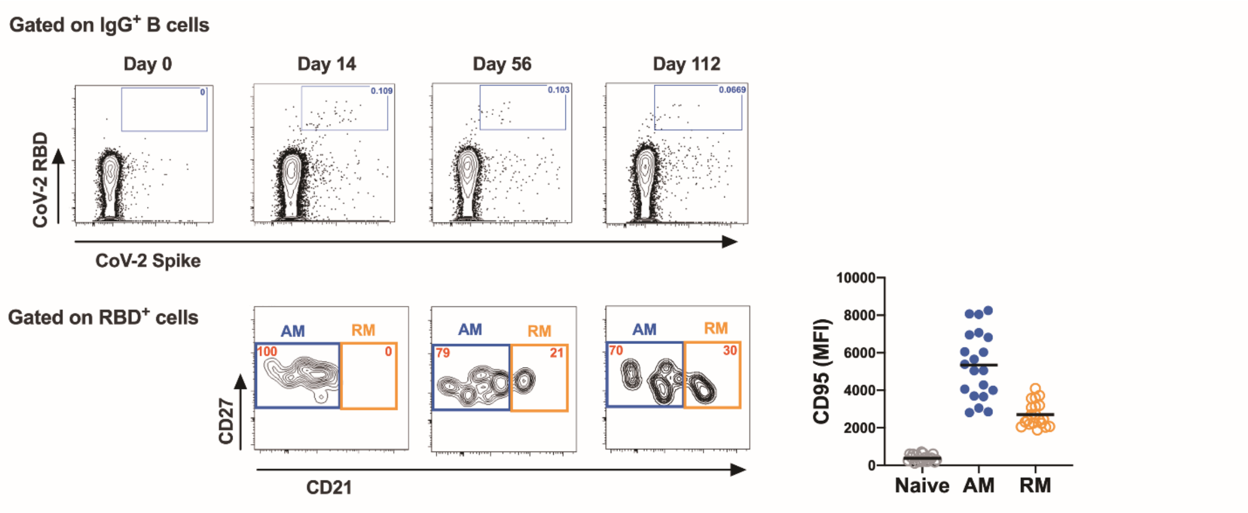


**Fig. S3. Dynamics of activated and resting memory B cells following single-shot Ad26.COV2.S vaccination.** Representative flow cytometry plots show PBMCs from one vaccinated macaque on days 0, 14, 56, and 112 after vaccination gated on class-switched IgG+ B cells. The lower panels show activated memory (AM; blue) and resting memory (RM; orange) B cell populations gated on RBD-binding B cells. The geometric mean fluorescent intensity (MFI) of CD95 expression on naive B, AM B, and RM B cell subsets was measured. Bold horizontal lines reflect median values.

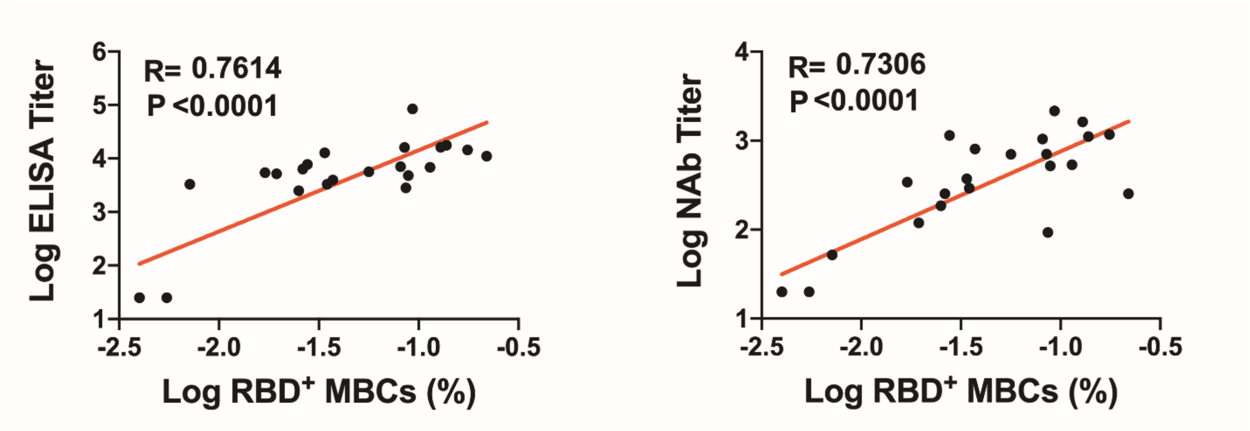


**Fig. S4. Correlations between vaccine-induced memory B cell response and antibody responses.** Correlations between SARS-CoV-2 RBD-specific memory B cells (MBCs) and binding or neutralizing antibody titers on day 28 following vaccination are shown. Red lines reflect the best linear fit relationship between these variables. P and R values reflect two-sided Spearman rank-correlation tests.

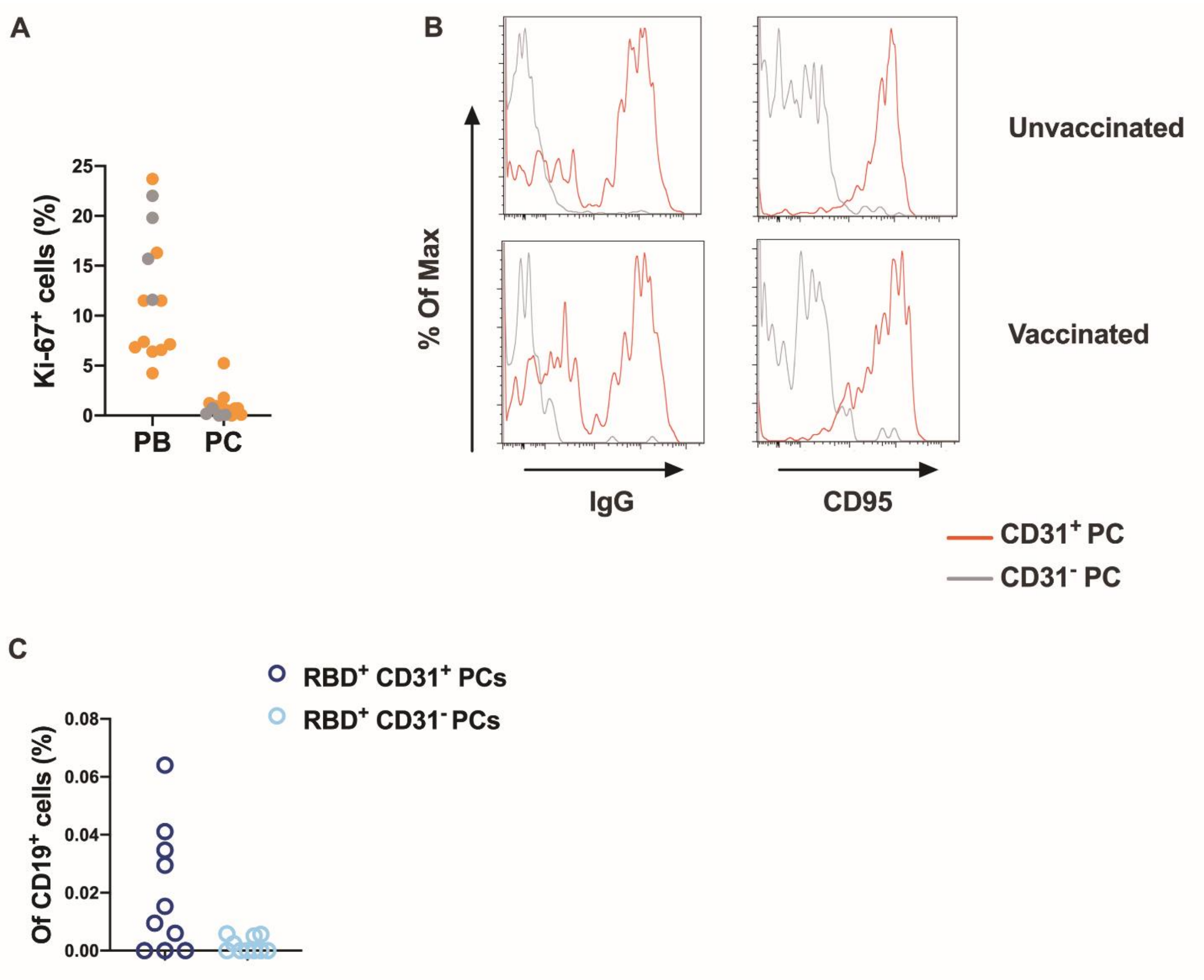


**Fig. S5. Plasma cell subsets in bone marrow cells.** (**A**) Expression of Ki-67 was measured on plasmablasts (PB) in PBMC and plasma cells (PC) in bone marrow from unvaccinated macaques (gray) and vaccinated macaques (orange). (**B**) Representative flow cytometry histograms of IgG and CD95 expression on $CD31^+$ and $CD31^-$ plasma cells from an unvaccinated and a vaccinated macaque are shown. (**C**) Frequencies of RBD-specific $CD31^+$ and $CD31^-$ plasma cells in $CD19^+$ B cells from vaccinated macaques are shown.

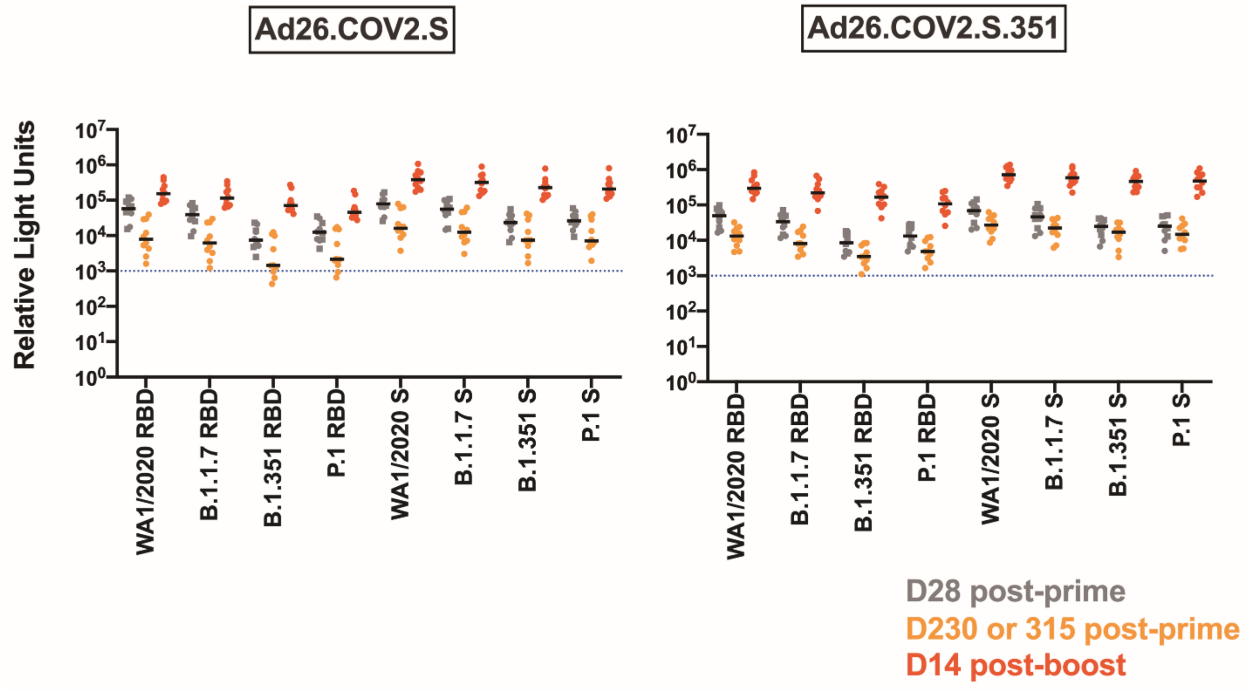


**Fig. S6. ECLA binding antibody responses following single-shot Ad26.COV2.S vaccination and boost immunization.** RBD- and spike (S) protein-specific binding antibody responses against the SARS-CoV-2 WA1/2020, B.1.1.7 (alpha), B.1.351 (beta), and P.1 (gamma) variants were assessed by electrochemiluminescence assays (ECLA) on day 28 and day 230 or 315 following initial vaccination and on day 14 after boost immunization with Ad26.COV2.S or Ad26.COV2.S.351. Bold horizontal lines reflect median values. Dotted lines reflect lower limits of quantitation.

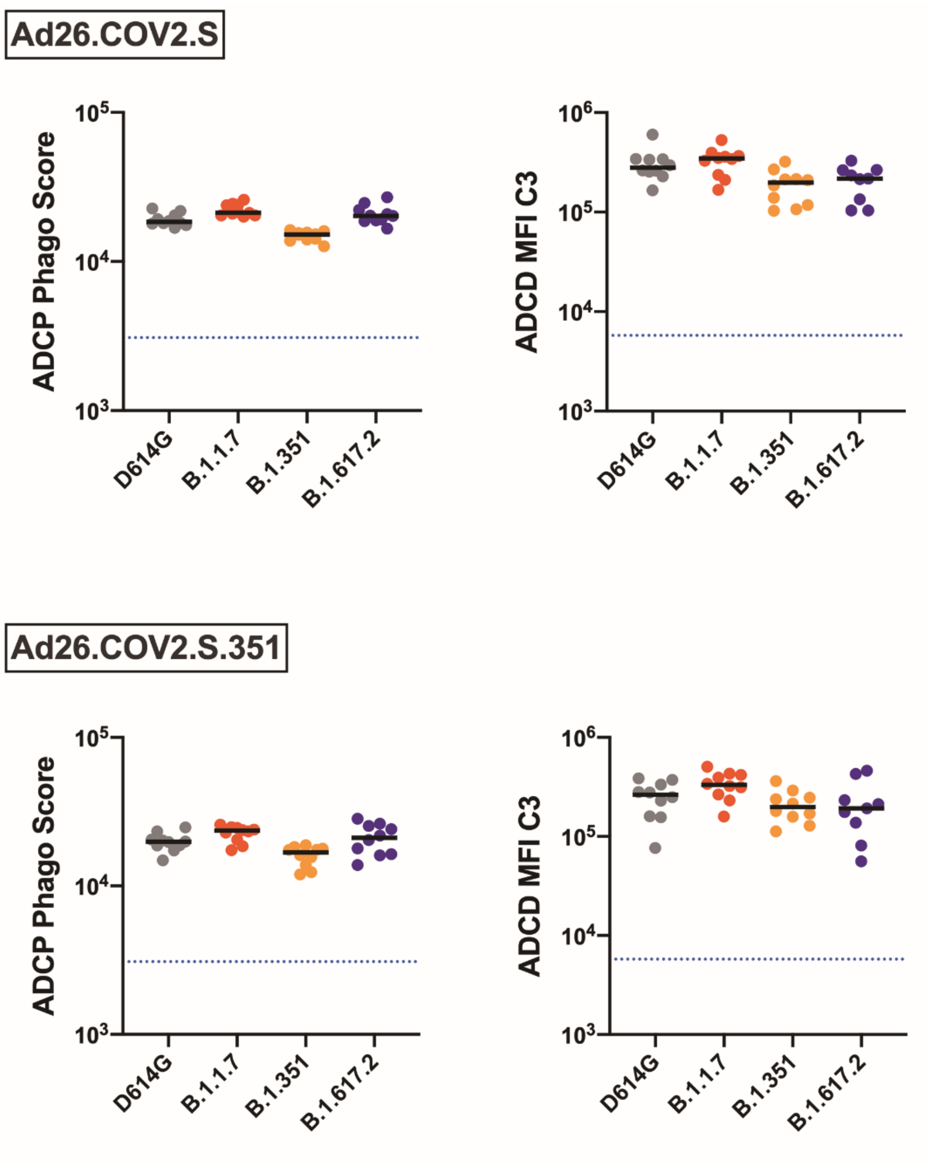


**Fig. S7. Antibody-dependent cellular phagocytosis (ADCP) and antibody-dependent complement deposition (ADCD) responses following boost immunization.** Spike protein-specific ADCP and ADCD responses against the SARS-CoV-2 D614G, B.1.1.7 (alpha), B.1.351 (beta), and B.1.617.2 (delta) variants were assessed on day 14 after boost immunization with Ad26.COV2.S or Ad26.COV2.S.351. Bold horizontal lines reflect median values. Dotted lines reflect lower limits of quantitation.

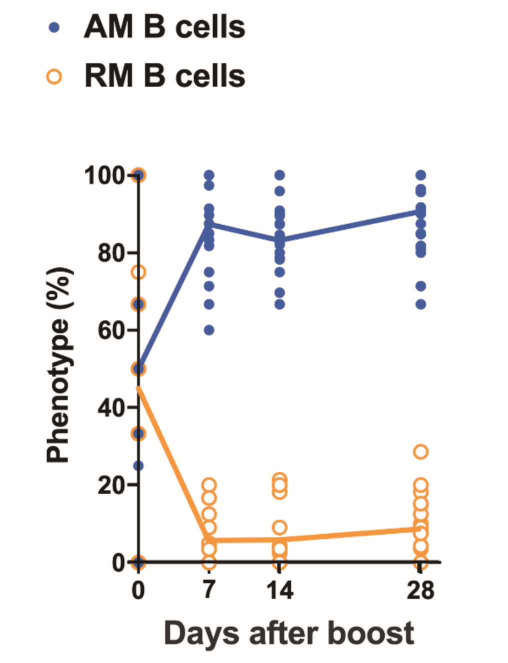


**Fig. S8. Dynamics of activated and resting memory B cells following boost immunization.** The proportions of SARS-CoV-2 RBD-specific activated memory (AM; blue) and resting memory (RM; orange) B cells in $IgG^+$ B cells were measured following boost immunization. Lines show median values.

**Supplementary Data Files**

Data File S1. Individual-level neutralizing antibody data.

Data File S2. Individual-level ELISA data.

Data File S3. Individual-level B cell flow cytometry data.

Data File S4. Individual-level ELISPOT data.

Data File S5. Individual-level electrochemiluminescence assay data.

Data File S6. Individual-level systems serology data.
