# Supplementary material for: A homologous or variant booster vaccine after Ad26.COV2.S immunization enhances SARS-CoV-2–specific immune responses in rhesus macaques
Source: Sci Transl Med. 2022 Mar 30;14(638):eabm4996. doi: 10.1126/scitranslmed.abm4996 (PMC9802654; doi:10.1126/scitranslmed.abm4996)
Supplement: Supplementary file 4 — MDAR Reproducibility Checklist [file scitranslmed.abm4996_mdar_reproducibility_checklist.zip › scitranslmed.abm4996_mdar_reproducibility_checklist.docx]

**Materials Design Analysis Reporting (MDAR)**

**Checklist for Authors**

The MDAR framework establishes a minimum set of requirements in transparent reporting applicable to studies in the life sciences (see Statement of Task: doi:10.31222/osf.io/9sm4x.). The MDAR checklist is a tool for authors, editors, and others seeking to adopt the MDAR framework for transparent reporting in manuscripts and other outputs. Please refer to the MDAR Elaboration Document for additional context for the MDAR framework.

**For all that apply, please note where in the manuscript the required information is provided.**

**Materials:**

| **Newly created materials** | **indicate where provided: page no/section/legend)** | **n/a** |
| --- | --- | --- |
| The manuscript includes a dedicated "materials availability statement" providing transparent disclosure about availability of newly created materials including details on how materials can be accessed and describing any restrictions on access. | Ad26.COV2.S.351, Ad26.COV.2, Methods |  |
|  |  |  |
| **Antibodies** | **indicate where provided: page no/section/legend)** | **n/a** |
| For commercial reagents, provide supplier name, catalogue number and [RRID](https://scicrunch.org/resources), if available. | Section Methods“B cell immunophenotyping” :  Mouse anti-human Ki-67 PerCP-cy5.5BD (Pharmingen Cat # 561284 RRID:AB_10611574); Goat anti-human IgD PE(Southern Biotec Cat # 2030-09 RRID:AB_2795630); Mouse anti-human CD138 PE-CF594 (BIOLEGEND Cat # 352320 RRID:AB_2687342 ); Mouse anti-human CD20 PE-Cy5 (BD Pharmingen Cat # 555624 RRID:AB_395990 ); Rat anti-human IRF4 PE Cy7 (Affymetrix Cat # 25-9858-82 RRID:AB_2573558 ); Mouse anti-human CD14 BV570 (BIOLEGEND Cat # 301832 RRID:AB_2563629); Mouse anti-human CD21 BV605 (BD Pharmingen Cat # 740395 RRID:AB_2740125 ); Mouse anti-human CD95 BV711(BIOLEGEND Cat # 305644 RRID:AB_2632623); Mouse anti-human CD80 BV786 (BD Pharmingen Cat # 564159 RRID:AB_2738631); Mouse anti-human IgM BUV395 (BD Pharmingen Cat # 563903 RRID:AB_2721269 ); Mouse anti-human CD27 BUV563 (BD Pharmingen Cat # 741366 RRID:AB_2870866); Mouse anti-human IgG BUV737 (BD Pharmingen Cat # 612819 RRID:AB_2870143 ); Mouse anti-human CD45 BUV805(BD Pharmingen Cat # 742055 RRID:AB_2871344 ); Goat anti-human IgA APC (FISHER/JACKSON Cat # 109-135-011 RRID:AB_2337689 ); Mouse anti-human CD11c Alexa700 (Affymetrix Cat # 56-0116-42 RRID:AB_10547281 ); Mouse anti-human CD123 Alexa700 (FISHER/NOVUS Cat # NB6001185AF700); Mouse anti-human CD7 Alexa700 (BD Pharmingen Cat # 561603 RRID:AB_10898348 ); Mouse anti-human CD3 APC-Cy7 (BD Pharmingen Cat # 557757 RRID:AB_396863);  Section Methods“ECLA”:  MesoScale Discovery Kits SARS-CoV-2 IgG Cat No: N05CA-1; Panels 11 and 13  Section Methods “ELISPOT”:  IFN-γ monoclonal antibody from BD Pharmigen (554699); SARS-CoV-2 peptides (21st Century Biochemicals; the variants peptides contain the WT backbone); Rabbit polyclonal anti-human IFN-γ Biotin from U-Cytech (CT243); Streptavidin-alkaline phosphatase from Southern Biotech (7105-04); Nitro-blue Tetrazolium Chloride/5-bromo-4-chloro 3 ‘indolyphosphate p-toludine salt (NBT/BCIP chromogen) (Fisher Scientific) (34042)  Section Methods “ELISA”:  Anti-macaque IgG HRP(NHPRR, Clone IB3) |  |
|  |  |  |
| **DNA and RNA sequences** | **indicate where provided: page no/section/legend)** | **n/a** |
| **Short novel DNA or RNA including primers, probes:** Sequences should be included or deposited in a public repository. |  | n/a |
|  |  |  |
| **Cell materials** | **indicate where provided: page no/section/legend** | **n/a** |
| **Cell lines:** Provide species information, strain. Provide accession number in repository **OR** supplier name, catalog number, clone number, **OR** RRID. | Section Methods “Pseudovirus-based virus neutralization assay”  HEK293T (ATCC)  Section Methods “System serology”  THP-1 cells (ATCC) |  |
| **Primary cultures:** Provide species, strain, sex of origin, genetic modification status. |  | n/a |
|  |  |  |
| **Experimental animals** | **indicate where provided: page no/section/legend)** | **n/a** |
| **Laboratory animals or Model organisms:** Provide species, strain, sex, age, genetic modification status. Provide accession number in repository **OR** supplier name, catalog number, clone number, **OR** RRID. | Section Methods “Animals and study design”：  24 outbred Indian-origin adult male and female rhesus macaques (Macaca mulatta) ages 4-22 years old were randomly allocated to groups. All animals were housed at Bioqual, Inc. (Rockville, MD). |  |
| **Animal observed in or captured from the field:** Provide species, sex, and age where possible. |  | n/a |
|  |  |  |
| **Plants and microbes** | **indicate where provided: page no/section/legend)** | **n/a** |
| **Plants:** provide species and strain, ecotype and cultivar where relevant, unique accession number if available, and source (including location for collected wild specimens). |  | n/a |
| **Microbes:** provide species and strain, unique accession number if available, and source. |  |  |
|  |  |  |
| **Human research participants** | **indicate where provided: page no/section/legend) or state if these demographics were not collected** | **n/a** |
| If collected and within the bounds of privacy constraints report on age, sex and gender or ethnicity for all study participants. |  | n/a |

**Design:**

| **Study protocol** | **indicate where provided: page no/section/legend)** | **n/a** |
| --- | --- | --- |
| If study protocol has been pre-registered, provide DOI. For clinical trials, provide the trial registration number **OR** cite DOI. | Section Methods “Animals and study design”：  IACUC approval for animal studies |  |
|  |  |  |
| **Laboratory protocol** | **indicate where provided: page no/section/legend)** | **n/a** |
| Provide DOI **OR** other citation details if detailed step-by-step protocols are available. |  | n/a |
|  |  |  |
| **Experimental study design (statistics details)** | | |
| **For in vivo studies:** State whether and how the following have been done | **indicate where provided: page no/section/legend. If it could have been done, but was not, write not done** | **n/a** |
| Sample size determination | Section Methods “Animals and study design”：  Sample size and age criteria were determined based on the results of previous non-human primate studies. |  |
| Randomisation | Section Methods “Animals and study design”：  24 outbred Indian-origin adult male and female rhesus macaques (Macaca mulatta) ages 4-22 years old were randomly allocated to groups. |  |
| Blinding | Section Methods “Animals and study design”:  All immunologic studies were performed blinded. |  |
| Inclusion/exclusion criteria |  | n/a |
|  |  |  |
| **Sample definition and in-laboratory replication** | **indicate where provided: page no/section/legend** | **n/a** |
| State number of times the experiment was replicated in laboratory. |  | n/a |
| Define whether data describe technical or biological replicates. |  | n/a |
|  |  |  |
| **Ethics** | **indicate where provided: page no/section/legend** | **n/a** |
| **Studies involving human participants:** State details of authority granting ethics approval (IRB or equivalent committee(s), provide reference number for approval. |  | n/a |
| **Studies involving experimental animals:** State details of authority granting ethics approval (IRB or equivalent committee(s), provide reference number for approval. | Section Methods “Animals and study design”:  Animal studies were conducted in compliance with all relevant local, state, and federal regulations and were approved by the Bioqual Institutional Animal Care and Use Committee (IACUC). |  |
| **Studies involving specimen and field samples:** State if relevant permits obtained, provide details of authority approving study; if none were required, explain why. |  | n/a |
|  |  |  |
| **Dual Use Research of Concern (DURC)** | **indicate where provided: page no/section/legend** | **n/a** |
| If study is subject to dual use research of concern regulations, state the authority granting approval and reference number for the regulatory approval. |  | n/a |

**Analysis:**

| **Attrition** | **indicate where provided: page no/section/legend** | **n/a** |
| --- | --- | --- |
| Describe whether exclusion criteria were preestablished. Report if sample or data points were omitted from analysis. If yes report if this was due to attrition or intentional exclusion and provide justification. | Section Methods “Animals and study design”：  No data points were omitted from analysis |  |
|  |  |  |
| **Statistics** | **indicate where provided: page no/section/legend** | **n/a** |
| Describe statistical tests used and justify choice of tests. | Section Methods “Statistical analyses”：  Comparisons of immunologic data was performed using GraphPad Prism 8.4.2 (GraphPad Software). Comparison of data between groups was performed using two-sided Mann-Whitney tests or Wilcoxon signed-rank tests (for matched pairs). P values of less than 0.05 were considered significant. For differential expression gene (DEG) analyses, P values were corrected for multiple testing using the Benjamini-Hochberg (BH) method and a cut-off of 0.05. Pathway enrichment analysis was performed using R and was assessed using a false discovery rate (FDR) cut-off of 5%. Correlation analyses were performed using the cor.test R package, and statistical significance was assessed using two-sided Spearman rank-correlation tests. |  |
|  |  |  |
| **Data availability** | **indicate where provided: page no/section/legend** | **n/a** |
| For newly created and reused datasets, the manuscript includes a data availability statement that provides details for access or notes restrictions on access. |  | n/a |
| If newly created datasets are publicly available, provide accession number in repository **OR** DOI **OR** URL and licensing details where available. | Section Methods “RNA sequecing”and “Data Availability Statement”:  Raw fastq files were uploaded to NCBI Gene Expression Omnibus (GEO) database under identifier GSE193264. |  |
| If reused data is publicly available provide accession number in repository **OR** DOI **OR** URL, **OR** citation. |  | n/a |
|  |  |  |
| **Code availability** | **indicate where provided: page no/section/legend** | **n/a** |
| For all newly generated custom computer code/software/mathematical algorithm or re-used code essential for replicating the main findings of the study, the manuscript includes a data availability statement that provides details for access or notes restrictions. |  | n/a |
| If newly generated code is publicly available, provide accession number in repository, **OR** DOI **OR** URL and licensing details where available. State any restrictions on code availability or accessibility. |  | n/a |
| If reused code is publicly available provide accession number in repository **OR** DOI **OR** URL, **OR** citation. |  | n/a |

**Reporting**

MDAR framework recommends adoption of discipline-specific guidelines, established and endorsed through community initiatives. Journals have their own policy about requiring specific guidelines and recommendations to complement MDAR.

| **Adherence to community standards** | **indicate where provided: page no/section/legend** | **n/a** |
| --- | --- | --- |
| State if relevant guidelines (e.g., ICMJE, MIBBI, ARRIVE) have been followed, and whether a checklist (e.g., CONSORT, PRISMA, ARRIVE) is provided with the manuscript. |  | n/a |
